# Supplementary material for: Association of impaired kidney function with mortality in rural Uganda: results of a general population cohort study
Source: BMJ Open. 2022 Apr 26;12(4):e051267. doi: 10.1136/bmjopen-2021-051267 (PMC9045120; doi:10.1136/bmjopen-2021-051267)
Supplement: Supplementary data [file bmjopen-2021-051267supp001.pdf]

Supplementary Appendix for

"Association of impaired kidney function with mortality in rural Uganda: results of a general population cohort study"

Kalyesubula R, *et al. BMJ Open* 2022; 12:e051267. doi: 10.1136/bmjopen-2021-051267

Supplementary Table 1. Comparison between participants included and excluded from the main analysis

Variable	Participants with complete data included in fully adjusted model N=3102	Participants excluded from the final model N=2567	P-value
**eGFR (ml/min/1.73m^2)**			
≥90	2231 (71·9)	2332 (90.5)	
60-89	792 (25·6)	230 (8.9)	
45-59	63 (2·0)	10 (0.4)	
<45	16 (0·5)	4 (0·2)	<0.001
**Gender**			
Male	1150 (37·1)	1079 (41.9)	
Female	1952 (62·9)	1497 (58.1)	<0.001
**Age groups, N (%)**			
<35	733 (23·6)	1880 (73.0)	
35-44	772 (24·9)	356 (13.8)	
45-54	682 (22·0)	160 (6.2)	
55-64	451 (14·5)	89 (3.5)	
65-75	291 (9·4)	52 (2.0)	
>75	173 (5·6)	39 (1.5)	<0.001
**Age (years), median (IQR)**	45 (35-57)	24 (19-36)	<0.001
**HIV status, **N (%)**			
Negative	2756 (88·8)	2362 (91.7)	
Positive	346 (11·2)	205 (8.0)	
Missing	··	9 (0·3)	<0.001
**BMI classification, (kg/m^2) # N (%)**			
Underweight (<18·5)	415 (13·4)	250 (9.7)	
Normal weight (18·5–24·99)	2029 (65·4)	1850 (71.8)	
Overweight (25·0–29·99)	484 (15·6)	250 (9.7)	
Obese (>30·0)	174 (5·6)	73 (2.8)	
Missing	··	153 (5.9)	<0.001
**Diabetes mellitus, ## N (%)**			
No	3025 (97·5)	866 (33.6)	
Yes	77 (2·5)	8 (0.3)	
Missing	··	1702 (66.1)	<0.001
**Hypertension, ### N (%)**			
Normal	2583 (83·3)	831 (32.3)	
Hypertensive	519 (16·7)	65 (2.5))	
Missing	··	1680 (65.2)	<0.001
**Smoking Status, N (%)**			
Not current smokers	2757 (88·9)	860 (33.4)	
Non-daily smokers	79 (2·6)	17 (0.7)	
Daily smokers	266 (8·5)	18 (0.7)	
Missing	··	1681 (65.3)	<0.001
**Alcohol Consumption, N (%)**			
Regular alcohol	1601 (51·6)	736 (28.6)	
Infrequent/no alcohol	1501 (48·4)	160 (6.2)	
Missing	··	1680 (65.2)	<0.001
**Currently married, N(%)**			
Married	978 (31·5)	369 (14.3)	
Single/widowed	2124 (68·5)	948 (36.8)	
Missing	··	1259 (48.9)	<0.001

#Body Mass Index (BMI) Classification according to WHO (weight/height^2: kg/m^2)
##Diabetes mellitus defined as HBA1C >6.5%, previously diagnosed with diabetes mellitus or being on treatment for diabetes
###Hypertension was defined as having a systolic BP ≥90 mmHg, diastolic BP ≥140 mmHg or being on treatment for hypertension

Kalyesubula R, *et al. BMJ Open* 2022; 12:e051267. doi: 10.1136/bmjopen-2021-051267

Supplementary Table 2. Baseline characteristics of participants by five-level categories of kidney function in the general population cohort (n=5,678)

	Estimated glomerular filtration rate (mls/min/1.73m^2)					
Variable	**≥120**<br>**N%**<br>**1966 (34.6)**	**90-119**<br>**N%**<br>**2597 (45.7)**	**60-89**<br>**N%**<br>**1022 (18.0)**	**45-59**<br>**N%**<br>**73 (1.3)**	**<45**<br>**N%**<br>**20 (0.4)**	**Total**
**Sex**						
Male	709 (36·1)	1144 (44·1)	343 (33·6)	26 (35·6)	7 (35·0)	2229 (39·3)
Female	1257 (63·9)	1453 (56·0)	679 (66·4)	47 (64·4)	13 (65·0)	3449 (60·7)
**Age (years), median (IQR)**	22 (19-28)	41 (33-51)	56 (45-70)	66 (58-76)	68 (42-76)	36 (24-50)
**Age groups, N (%)**						
<35	1784 (90·7)	747 (28·8)	76 (7·4)	3 (4·1)	3 (15·0)	2613 (46·0)
35-44	158 (8·0)	788 (30·3)	176 (17·2)	4 (5·5)	2 (10·0)	1128 (19·9)
45-54	17 (0·9)	602 (23·2)	210 (20·6)	10 (13·7)	3 (15·0)	842 (14·8)
55-64	5 (0·3)	306 (11·8)	214 (20·9)	14 (19·2)	1 (5·0)	540 (9·5)
65-75	2 (0·1)	131 (5·0)	186 (18·2)	19 (26·0)	5 (25·0)	343 (6·0)
>75	0 (0·0)	23 (0·9)	160 (15·7)	23 (31·5)	6 (30·0)	212 (3·7)
**HIV status, N (%)**						
Negative	1836 (93·4)	2298 (88·5)	901 (88·2)	70 (96·0)	13 (65·0)	5118 (90·1)
Positive	127 (6·5)	297 (11·4)	117 (11·5)	3 (4·1)	7 (35·0)	551 (9·7)
Missing	3 (0·2)	2 (0·1)	4 (0·4)	0 (0·0)	0 (0·0)	9 (0·2)
**BMI classification, # (kg/m^2) N (%)**						
Underweight (<18·5)	185 (9·4)	319 (12·3)	143 (14·0)	14 (19·2)	4 (20·0)	665 (11·7)
Normal weight (18·5–<25)	1429 (72·7)	1772 (68·2)	623 (61·0)	41 (56·2)	14 (70·0)	3879 (68·3)
Overweight (25·0–<30)	195 (9·9)	357 (13·8)	166 (16·2)	14 (19·2)	2 (10·0)	734 (12·9)
Obese (≥30·0)	46 (2·3)	121 (4·7)	77 (7·6)	3 (4·1)	0 (0·0)	247 (4·4)
Missing	111 (5·7)	28 (1·1)	13 (1·3)	1 (1·4)	0 (0)	153 (2·7)
**Diabetes mellitus, ## N (%)**						
No	1096 (55·8)	1902 (73·2)	815 (79·8)	62 (84·9)	16 (80·0)	3926 (68·5)
Yes	13 (0·7)	49 (1·9)	20 (2·0)	3 (4·1)	0 (0·0)	85 (1·5)
Missing	857 (43·6)	646 (24·9)	187(18·3)	8 (11·0)	4 (20·0)	1702 (30·0)
**Hypertension, ### N (%)**						
Normal	1064 (54·1)	1692 (65·2)	614 (60·1)	35 (47·9)	9 (45·0)	3414 (60·1)
Hypertensive	48 (2·4)	271 (10·4)	228 (22·3)	30 (41·1)	7 (35·0)	584 (10·3)
Missing	854 (43·4)	634 (24·4)	180 (17·6)	8 (11·0)	4 (20·0)	1680 (29·6)
**Smoking Status, N (%)**						
Not current smokers	1061 (54·0)	1740 (67·0)	748 (73·2)	55 (75·3)	13 (65·0)	3617 (63·7)
Non-daily smokers	13 (0·7)	51 (2·0)	30 (2·9)	1 (1·4)	1 (5·0)	96 (1·7)
Daily smokers	38 (1·9)	171 (6·6)	64 (6·3)	9 (12·3)	2 (10·0)	284 (5·0)
Missing	854 (43·4)	635 (24·5)	180 (17·6)	8 (11·0)	4 (20·0)	1681 (29·6)
**Alcohol Consumption, N (%)**						
No	854 (43·4)	1057 (40·7)	393 (38·5)	29 (39·7)	4 (20·0)	2337 (41·2)
Yes	258 (13·1)	906 (34·9)	449 (43·9)	36 (49·3)	12 (60·0)	1661 (29·3)
Missing	854 (43·4)	634 (24·4)	180 (17·6)	8 (11·0)	4 (20·0)	1680 (29·6)
**Marital status, N (%)**						
Married	162 (8·2)	691 (26·6)	444 (43·4)	37 (50·7)	13 (65·0)	1347 (23·7)
Single/widowed	888 (45·2)	1608 (61·9)	536 (52·5)	33 (45·2)	7 (35·0)	3072 (54·1)
Missing	916 (46·6)	298 (11·5)	42 (4·1)	3 (4·1)	0 (0·0)	1259 (22·2)

#Body Mass Index (BMI) Classification according to WHO (weight/height^2: kg/m^2)
##Diabetes mellitus defined as HBA1C >6.5%, previously diagnosed with diabetes mellitus or being on treatment for diabetes
###Hypertension was defined as having a systolic BP ≥90 mmHg, diastolic BP ≥140 mmHg or being on treatment for hypertension

Supplementary Table 3. Results of age-sex and fully adjusted regression models for the association between five category kidney function and mortality in the general population cohort

	**Age and sex adjusted**		**Fully adjusted#**	
	**HR (95% CI)** **N=5678**	**P-value**	**HR (95% CI)** **N=3102**	**P-value**
**eGFR (mls/min/1.73m²)**				
≥120	2·68 (1·47-4·87)		1·65 (0·61-4·44)	
90-119	**reference**		**reference**	
60-89	1·14 (0·73-1·77)		1·23 (0·73-2·06)	
45-59	1·67 (0·77-3·60)		1·90 (0·79-4·52)	
<45	5·58 (2·36-13·24)	<0·001	6·06 (2·25-16·37)	0·005
**Age (years)**	1·06 (1·05-1·07)	<0·001	1·06 (1·04-1·08)	<0·001
**Sex**				
Male	**reference**		**reference**	
Female	0·50 (0·36-0·70)	<0·001	0·71 (0·42-1·19)	0·19
**HIV status**				
Positive			1·69 (0·89-3·23)	0·11
Negative				
**Hypertension**				
Normotensive			**reference**	
Hypertensive			0·82 (0·51-1·33)	0·42
**Diabetes mellitus**				
No			**reference**	
Yes			1·11 (0·34-3·56)	0·87
**BMI classification**				
Under weight			1·70 (1·09-2·66)	
Normal weight			**reference**	
Overweight			0·20 (0·05-0·84)	
Obese			0·35 (0·05-2·58)	0·006
**Marital status**				
Married			0·77 (0·48-1·22)	0·27
Single/widowed			**reference**	
**Alcohol consumption**				
Regular alcohol			1·50 (0·92-2·45)	0·11
Infrequent/no alcohol			**reference**	
**Smoking status**				
Not current smokers			**reference**	
Non-daily smokers			1·23 (0·51 - 3·00)	
Daily smokers			1·63 (0·92 - 2·86)	0·24

Fully Adjusted Hazard Ratios adjusted for age, sex, HIV status, hypertension, diabetes mellitus, BMI, marital status, alcohol use and smoking.

Supplementary Figure 1: Hazard ratios and 95% CI for the fully adjusted associations of baseline eGFR (with eGFR >120mls/min as a separate category) and mortality in a rural Uganda population cohort

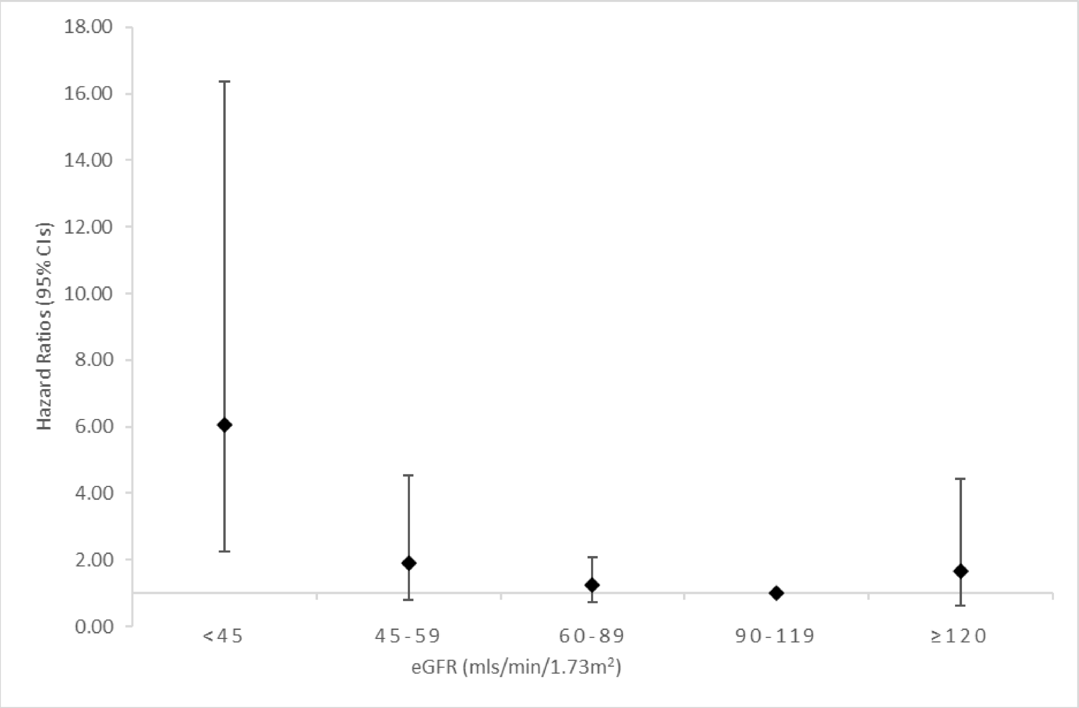

Kalyesubula R, *et al. BMJ Open* 2022; 12:e051267. doi: 10.1136/bmjopen-2021-051267
