# Supplementary material for: Association of impaired kidney function with mortality in rural Uganda: results of a general population cohort study
Source: BMJ Open. 2022 Apr 26;12(4):e051267. doi: 10.1136/bmjopen-2021-051267 (PMC9045120; doi:10.1136/bmjopen-2021-051267)
Supplement: Author's manuscript [file bmjopen-2021-051267.draft_revisions.pdf]

BMJ Open is committed to open peer review. As part of this commitment we make the peer review history of every article we publish publicly available.

When an article is published we post the peer reviewers' comments and the authors' responses online. We also post the versions of the paper that were used during peer review. These are the versions that the peer review comments apply to.

The versions of the paper that follow are the versions that were submitted during the peer review process. They are not the versions of record or the final published versions. They should not be cited or distributed as the published version of this manuscript.

BMJ Open is an open access journal and the full, final, typeset and author-corrected version of record of the manuscript is available on our site with no access controls, subscription charges or pay-per-view fees (http://bmjopen.bmj.com).

If you have any questions on BMJ Open's open peer review process please email info.bmjopen@bmj.com

BMJ Open

Association of impaired kidney function with mortality in rural Uganda: results of a general population cohort study

Journal:	*BMJ Open*
Manuscript ID	bmjopen-2021-051267
Article Type:	Original research
Date Submitted by the Author:	23-Mar-2021
Complete List of Authors:	Kalyesubula, Robert; London School of Hygiene and Tropical Medicine Faculty of Epidemiology and Population Health; Makerere University College of Health Sciences, Physiology and Internal Medicine<br>Sekitoleko, Isaac; MRC/UVRI and LSHTM Research Unit<br>Tomlin, Keith; London School of Hygiene and Tropical Medicine Faculty of Epidemiology and Population Health<br>Hansen, Christian; London School of Hygiene & Tropical Medicine, Infectious Diseases Epidemiology<br>Ssebunya, Billy; MRC/UVRI and LSHTM Research Unit<br>Makanga, Ronald; MRC/UVRI and LSHTM Research Unit<br>Mbonye, Moses Kwizera; MRC/UVRI and LSHTM Research Unit<br>Seeley, Janet; London School of Hygiene and Tropical Medicine, Department of Global Health &Development<br>Smeeth, Liam; London School of Hygiene and Tropical Medicine Faculty of Epidemiology and Public Health<br>Newton, Robert; University of York Department of Health Sciences; Medical Research Council/Uganda Virus Research Institute<br>Tomlinson, Laurie; London School of Hygiene & Tropical Medicine, Epidemiology and Population Health
Keywords:	Nephrology < INTERNAL MEDICINE, PUBLIC HEALTH, EPIDEMIOLOGY

SCHOLARONE™
Manuscripts

I, the Submitting Author has the right to grant and does grant on behalf of all authors of the Work (as defined in the below author licence), an exclusive licence and/or a non-exclusive licence for contributions from authors who are: i) UK Crown employees; ii) where BMJ has agreed a CC-BY licence shall apply, and/or iii) in accordance with the terms applicable for US Federal Government officers or employees acting as part of their official duties; on a worldwide, perpetual, irrevocable, royalty-free basis to BMJ Publishing Group Ltd ("BMJ") its licensees and where the relevant Journal is co-owned by BMJ to the co-owners of the Journal, to publish the Work in this journal and any other BMJ products and to exploit all rights, as set out in our licence.

The Submitting Author accepts and understands that any supply made under these terms is made by BMJ to the Submitting Author unless you are acting as an employee on behalf of your employer or a postgraduate student of an affiliated institution which is paying any applicable article publishing charge ("APC") for Open Access articles. Where the Submitting Author wishes to make the Work available on an Open Access basis (and intends to pay the relevant APC), the terms of reuse of such Open Access shall be governed by a Creative Commons licence – details of these licences and which Creative Commons licence will apply to this Work are set out in our licence referred to above.

Other than as permitted in any relevant BMJ Author's Self Archiving Policies, I confirm this Work has not been accepted for publication elsewhere, is not being considered for publication elsewhere and does not duplicate material already published. I confirm all authors consent to publication of this Work and authorise the granting of this licence.

For peer review only

Association of impaired kidney function with mortality in rural Uganda: results of a general population cohort study

Robert Kalyesubula[#1,2 3], Isaac Sekitoleko[3], Keith Tomlin[1], Christian Hansen[1,2], Billy Ssebunya[3], Ronald Makanga[3], Moses Kwizera Mbonye[3], Janet Seeley[1,3], Liam Smeeth[1], Robert Newton[3,4], Laurie A. Tomlinson[1]

Affiliations

1. London School of Hygiene and Tropical Medicine, London, UK
2. Makerere University College of Health Sciences, Kampala, Uganda
3. MRC/UVRI and LSHTM Research Unit, Entebbe, Uganda
4. University of York, York, UK

[#]Corresponding author

Dr Robert Kalyesubula

MRC/UVRI & LSHTM Research Unit, Entebbe, Uganda

Email: rkalyesubula@gmail.com

Word Count: 3250 words

For peer review only

For peer review only - http://bmjopen.bmj.com/site/about/guidelines.xhtml

Abstract

Objectives To determine the association between baseline kidney function and subsequent all-cause mortality.

Design and settings A general population-based cohort (GPC) study from rural Uganda.

Participants 5,678 participants aged 18 years and above with baseline estimated glomerular filtration rate (eGFR) recruited between 2011-2014 and followed up to March 2019.

Primary and secondary outcome measures The associations between baseline eGFR and all-cause mortality was determined using multivariable cox regression.

Results The median age of the participants at baseline was 36 years (IQR 24-50), 60.7% were female, 14.6% hypertensive, 9.7% HIV-positive and 1.8% diabetic. We registered 140 deaths with a median follow-up of 5.0 years. Adjusting for age and sex, HIV, hypertension, diabetes, BMI, marital status, and alcohol and tobacco use participants with eGFR ≤45 mls/min/1.73m^2 had six-fold higher mortality compared to those with eGFR ≥90mls/min/1·73m^2 (HR 6·12 (95% CI 2.27-16.45)) with strong evidence of a linear trend for risk of mortality as renal function declined (P<0.001).

Conclusion In a prospective cohort with high rates of follow-up we found that baseline kidney function was associated with subsequently increased mortality in a graded manner. Improved understanding of the determinants of kidney disease and its progression are needed in order to inform interventions for prevention and treatment.

For peer review only - http://bmjopen.bmj.com/site/about/guidelines.xhtml

Strengths and limitations of this study

- This is a large well-established population cohort with robust standardised procedures for detailed measurements of covariates such as kidney function and blood pressure, and creatinine was measured according to recommended international standards.
- We had a high participation and retention in the study within the local community and regular reporting of mortality and migration leading to limited loss to follow-up.
- To our knowledge this is the first time that a long-term prospective association between baseline impaired kidney function and mortality in an adult general population of this size has been demonstrated in sub-Saharan Africa.
- Outward migration by predominantly younger participants and use of a single measurement of creatinine without microalbuminuria may have led to a degree of bias.
- Information such as smoking, diabetes and blood pressure were not measured in both the 2011 and 2014 surveys of the GPC so our complete case analysis led to reduction in power for the fully-adjusted model.

For peer review only

For peer review only - http://bmjopen.bmj.com/site/about/guidelines.xhtml

Introduction

Chronic kidney disease (CKD) affects approximately one in every ten adults in high-income countries and is strongly associated with morbidity and mortality [1 2]. The leading causes of death among people with CKD include cardiovascular disease and infections while a proportion progress to end-stage kidney disease (ESKD) [3 4]. In high-income countries, ESKD is a chronic disease that can be managed with dialysis or kidney transplantation [5]. For low-income countries such as Uganda there is very limited access to kidney replacement therapies and most patients with ESKD die prematurely [6 7]. Compounding the challenge of managing kidney disease in sub-Saharan Africa (SSA) is that population prevalence estimates are limited [1], biochemical methods of measuring kidney function have been suboptimal [8], and the appropriate way to estimate kidney function is uncertain, since equations developed in high-income countries have not been validated for SSA [8-11]. In order to determine the value of developing health services to measure and manage kidney disease, we need to understand the relative importance of kidney function in determining prognosis compared to other chronic diseases in this setting. At present no population-based studies have examined the association between kidney function and mortality in sub-Saharan Africa. Therefore, we sought to determine the association between baseline kidney function and all-cause mortality in a large, well-described general population cohort of people in rural Uganda from 2011-2019.

Methods

Study design and setting

This was a prospective general population cohort (GPC) of people in Kyamulibwa, a rural community 132 kilometres from Kampala, the capital of Uganda. The GPC was established in 1989 by the Medical Research Council (MRC) UK and the Uganda Virus Research Institute (UVRI) to study the epidemiology of HIV infection. Subsequently, other diseases including non-communicable diseases have been examined in the same cohort. The population consists of rural subsistence farmers with a

For peer review only - http://bmjopen.bmj.com/site/about/guidelines.xhtml

few peri-urban dwellers and is similar to the broader rural populations of Uganda which constitute about 76% of the country[12]. This is an open cohort with new births, migrations (inward and outward) and deaths recorded regularly by community volunteers through the annual census. The participant response rate is high, at 95% [13]. Once every two years a survey is conducted to determine diseases and outcomes of interest. In 2011-12 and 2014-15, the major foci were diabetes, hypertension, obesity and chronic kidney disease. We collected data on subsequent mortality for participants who had a baseline creatinine measured in either 2011-2012 or 2014-2015.

Patient and Public Involvement in Research

This research was carried out with participation from members of the public of Kyamulibwa. As a research centre we have a community advisory board (CAB) which gets involved from inception of the studies to their implementation. The community was consulted on the priorities of research for the area in 2012 and non-communicable disease were identified as one of the key areas of research. Through seminars and face to face meetings community members were involved in the development of the key questions and outcome measures of the study on kidney disease. We developed simple tools on how the kidneys work and conducted several awareness campaigns in the villages to explain and get more input from the participants and other members of the community. We also undertook a qualitative study to understand the way community members appreciated kidney disease and this work has been published elsewhere[14] . Plans to disseminate our work involve sharing results with the community of Kyamulibwa as well as presentation to both national and international forum.

Participants

We identified potential participants above 18 years who had been recruited from household visits to a homestead in the community for creatinine analysis [15]. All participants had given informed consent for specimen storage and future use of their stored samples.

For peer review only - http://bmjopen.bmj.com/site/about/guidelines.xhtml

Variables

We extracted baseline demographic characteristics including age, sex, tribe, home address, socioeconomic status and maximum level of education. We also collected information on smoking, alcohol use, exercise patterns and diet in the census rounds of 2011-12 *or* 2014-15 (thus this information was not available for all participants for both rounds). We used participants' height and weight to determine their body mass index (BMI) using weight (kg) /height^2 (m^2). Participants with systolic blood pressure ≥140mmHg and/or diastolic blood pressures ≥90mmHg and those who were on treatment for hypertension were classified as hypertensive.

We also analysed blood for creatinine levels, hepatitis B, hepatitis C, HIV-infection and cholesterol levels. Diabetes mellitus was defined as having HbA1C >6.5%, being previously diagnosed with diabetes, or being on current treatment for diabetes. Detailed descriptions of these measurements have been published elsewhere [15]. Creatinine was measured using the enzymatic method traceable to an isotope dilution mass spectrometry method and 93.5% of all participants had a valid creatinine result (6.5% had no blood samples taken, usually because consent for this was declined) [15 16]. We calculated eGFR based on the CKD-Epi-creatinine equation without adjustment for ethnicity [5] and classified impaired kidney function in categories analogous to those used to define CKD stages [17]. We collected data on mortality through registers updated monthly through reports from community health workers (verified by verbal autopsy conducted in accordance with standard guidelines by WHO [18]) and cross-checked via annual census.

Statistical Analysis

We conducted a complete-case analysis with regard to baseline creatinine measurements and also excluded participants with missing data regarding outcome or with probable linkage errors. We described the population characteristics according to pre-defined categories of eGFR. We used multivariable Cox modelling to determine the Hazard Ratio (HR) for mortality for each category of eGFR. We tested the proportional hazards assumption using log-minus-log survival plots and visual

For peer review only - http://bmjopen.bmj.com/site/about/guidelines.xhtml

inspection of Kaplan-Meier curves. Due to missing data for some covariates (such as smoking and alcohol use) which were not collected in *both* the 2011 and 2014 surveys, we present models adjusted for age and sex, and then additionally for baseline HIV status, hypertension, diabetes mellitus, BMI, smoking, alcohol and marital status. Confounders were decided *a priori* based on research regarding associations of chronic conditions with eGFR in this population [15] and after investigation of co-linearity and data sparsity. We present the rate of mortality per 1,000 person-years, and report HR and 95% confidence intervals (CIs).

Because HIV is common in this population and has been associated with increased risk of both renal impairment and mortality, we examined potential interaction between eGFR and HIV status in the association with mortality by fitting an interaction term in the final adjusted model. In additional analysis, to examine for a 'J-shaped curve' in the association between eGFR and mortality we repeated the main analysis after re-categorisation of eGFR with those ≥120mls/min/1.73m^2 as a separate group. We analysed all data using STATA version 15.0 (StataCorp, College Station, TX, USA) statistical software.

Ethics

All study participants gave written informed consent to participate in the study. The study was approved by Uganda Virus Research Institute Research and Ethics Committee (UVRI-REC-#HS 1978), the Uganda National Council for Science and Technology (UNCST-#SS 4283) and the London School of Hygiene and Tropical Medicine Observational / Interventions Research Ethics Committee (LSHTM Ethics #21802)

Role of the funding source

The funders had no role in study design, data collection and analysis, decision to publish or preparation of the manuscript.

For peer review only - http://bmjopen.bmj.com/site/about/guidelines.xhtml

Results

We included 5,678 participants with kidney function and mortality data available for the analysis (**Figure 1**). The median age of the participants was 36 years (interquartile range (IQR) 24-50 years) with most being female (60.7%) and of normal weight (70.2%) while 9.7% were HIV-positive. The majority (90.5%) of the participants were non-smokers and 58.5% did not consume alcohol. 14.6% were hypertensive and 1.8% of the participants were diabetic (**Table 1**). Comparison of characteristics with included participants showed that those with missing data were younger but otherwise there were no important differences (**Supplementary Table 1**). We also included the baseline characteristics of participants by five-level stages of kidney function including eGFR > 120mls/min/1.73m^2 (**Supplementary Table 2**).

During follow-up there were 140 deaths with a median follow-up of 5.0 years (IQR 3.7-6.0). The incidence rate of death for the participants enrolled in the study was 4 deaths per 1000 person-years at risk (PYAR). For participants with eGFR >90mls/min/1.73m^2 the incident rate was about 3 deaths per 1000 (PYAR) person-years at risk and for those <60mls/min/1.73m^2 it was 27 deaths per 1000 PYAR. During follow-up, 26.4% (1,500/5,678) of participants migrated and 3.0% (169/5,678) were lost to follow-up.

For peer review only

Table 1. Baseline characteristics of participants categorised by level of kidney function in the general population cohort (N=5,678)

	Estimated glomerular filtration rate (mls/min/1.73m²)				
Variable	**≥90**	**60-89**	**45-59**	**<45**	**Total**
**Sex**					
Male	1853 (40.6)	343 (33.6)	26 (35.6)	7 (35.0)	2229 (39.3)
Female	2710 (59.4)	679 (66.4)	47 (64.4)	13 (65.0)	3449 (60.7)
**Age (years), median (IQR)**	32 (22-44)	56 (45-70)	66 (58-76)	68 (42-76)	36 (24-50)
**Age groups, N (%)**					
<35	2531 (55.5)	76 (7.4)	3 (4.1)	3 (15.0)	2613 (46.0)
35-44	946 (20.7)	176 (17.2)	4 (5.5)	2 (10.0)	1128 (19.9)
45-54	619 (13.6)	210 (20.6)	10 (13.7)	3 (15.0)	842 (14.8)
55-64	311 (6.8)	214 (20.9)	14 (19.2)	1 (5.0)	540 (9.5)
65-75	133 (2.9)	186 (18.2)	19 (26.0)	5 (25.0)	343 (6.0)
>75	23 (0.5)	160 (15.7)	23 (31.5)	6 (30.0)	212 (3.7)
**Marital status, N (%)**					
Single/widowed	853 (18.7)	444 (43.4)	37 (50.7)	13 (65.0)	1347 (23.7)
Married	2496 (54.7)	536 (52.5)	33 (45.2)	7 (35.0)	3072 (54.1)
Missing	1214 (26.6)	42 (4.1)	3 (4.1)	0 (0.0)	1259 (22.2)
**HIV status, N (%)**					
Negative	4134 (90.6)	901 (88.2)	70 (95.9)	13 (65.0)	5118 (90.1)
Positive	424 (9.3)	117 (11.5)	3 (4.1)	7 (35.0)	551 (9.7)
Missing	5 (0.1)	4 (0.4)	0 (0.0)	0 (0.0)	9 (0.2)
**Alcohol Consumption, N (%)**					
Infrequent/no alcohol	1911 (41.9)	393 (38.5)	29 (39.7)	4 (20.0)	2337 (41.2)
Regular alcohol	1164 (25.5)	449 (43.9)	36 (49.3)	12 (60.0)	1661 (29.3)
Missing	1488 (32.6)	180 (17.6)	8 (11.0)	4 (20.0)	1680 (29.6)
**Smoking Status, N (%)**					
Not current smokers	2801 (61.4)	748 (73.2)	55 (75.3)	13 (65.0)	3617 (63.7)
Non-daily smokers	64 (1.4)	30 (2.9)	1 (1.4)	1 (5.0)	96 (1.7)
Daily smokers	209 (4.6)	64 (6.3)	9 (12.3)	2 (10.0)	284 (5.0)
Missing	1489 (32.6)	180 (17.6)	8 (11.0)	4 (20.0)	1681 (29.6)
**Socioeconomic status**					
Lower	1064 (23.3)	289 (28.3)	21 (28.8)	3 (15.0)	1377 (24.3)
Middle	1059 (23.5)	257 (25.2)	23 (31.5)	7 (35.0)	1346 (23.7)
Upper	1029 (22.6)	272 (26.6)	21 (28.8)	4 (20.0)	1326 (23.4)
Missing	1411 (30.9)	204 (20.0)	8 (11.0)	6 (30.0)	1629 (28.7)
**Hypertension, N (%)**					
Normal	2756 (60.4)	614 (60.1)	35 (48.0)	9 (45.0)	3414 (60.1)
Hypertensive	319 (7.0)	228 (22.3)	30 (41.1)	7 (35.0)	584 (10.3)
Missing	1488 (32.6)	180 (17.6)	8 (11.0)	4 (20.0)	1680 (29.6)
**Diabetes mellitus, N (%)**					
No	2998 (65.7)	815 (79.8)	62 (84.9)	16 (100.0)	3891 (68.5)
Yes	62 (1.4)	20 (2.0)	3 (4.1)	0 (0.0)	85 (1.5)
Missing	1503 (32.9)	187 (18.3)	8 (11.0)	4 (20.0)	1702 (30.0)
**BMI classification, (kg/m²) N (%)**					
Underweight (<18.5)	504 (11.1)	143 (14.0)	14 (19.2)	4 (10.0)	665 (11.7)
Normal weight (18.5–24.99)	3201 (70.2)	623 (61.0)	41 (56.2)	14 (70.0)	3879 (68.3)
Overweight (25.0–29.99)	552 (12.1)	166 (16.2)	14 (19.2)	2 (10.0)	734 (12.9)
Obese (>30.0)	167 (3.7)	77 (7.5)	3 (4.1)	0 (0.0)	247 (4.4)
Missing	139 (3.1)	13 (1.3)	1 (1.4)	0 (0.0)	153 (2.7)

Missing data mainly arises due to different variables being collected in the 2011 and 2014 surveys of the GPC.

For peer review only

For peer review only - http://bmjopen.bmj.com/site/about/guidelines.xhtml

Age-sex and fully adjusted associations with mortality are shown in **Table 2** and **Figure 2**. In age and sex adjusted analyses, mortality was associated with poorer kidney function, being HIV positive, being unmarried or widowed, low BMI and regular smoking. In the fully-adjusted model, low kidney function remained associated with increased risk of mortality, (HR 6.12, 95% CI 2.27-16.45) for those with eGFR <45mls/min/1.73m^2 compared to those with eGFR ≥90mls/min/1.73m^2. The test for trend showed strong evidence (P<0.001) that the mortality rate increased progressively as category of baseline kidney function decreased. There was no evidence of an interaction between HIV and kidney function with risk of mortality in the fully adjusted model (P=0.672). In an additional analysis, when high eGFR was included as a separate category there was weak evidence of a 'J-shaped curve' (**Supplementary Table 3**). In age-sex adjusted analysis, baseline eGFR ≥120mls/min/1.73m^2 was associated with increased risk of mortality (HR 2.68, 95% CI 1.47-4.87) compared to the reference category of 90-119mls/min/1.73m^2 while in fully-adjusted analyses confidence intervals crossed the null (HR 1.65, 95% CI 0.61-4.44).

For peer review only

Table 2. Results of age-sex and fully-adjusted regression models for the association between kidney function and mortality in the general population cohort

	Age and sex adjusted		Fully adjusted#	
	**HR (95% CI)**	**P-value**	**HR (95% CI)**	**P-value**
**eGFR (mls/min/1.73m²)**				
≥90	**Reference**		**Reference**	
60-89	1.11 (0.73-1.71)		1.21 (0.73-2.03)	
45-59	1.72 (0.81-3.69)		1.91 (0.80-4.54)	
<45	5.97 (2.55-13.98)	<0.001	6.12 (2.27-16.45)	0.003
**Age (years)***	1.06 (1.05-1.07)	<0.001	1.05 (1.03-1.07)	<0.001
**Sex***				
Male	**Reference**		**Reference**	
Female	0.50 (0.36-0.70)	<0.001	0.70 (0.42-1.07)	0.188
**HIV status**				
Positive	2.29 (1.43-3.65)	<0.001	1.67 (0.87-3.18)	0.120
Negative	**Reference**		**Reference**	
**Hypertension**				
Normotensive	**Reference**		**Reference**	
Hypertensive	0.93 (0.60-1.43)	0.725	1.08 (0.58-2.01)	0.433
**Diabetes mellitus**				
No	**Reference**		**Reference**	
Yes	0.85 (0.27-2.67)	0.777	1.11 (0.35-3.57)	0.861
**BMI classification**				
Under weight	2.14 (1.49-3.09)		1.71 (1.10-2.67)	
Normal weight	**Reference**		**Reference**	
Over weight	0.21 (0.07-0.66)		0.20 (0.05-0.84)	
Obese	0.21 (0.03-1.50)	<0.001	0.35 (0.48-2.55)	0.006
**Marital status**				
Currently married	0.55 (0.37-0.81)	0.003	0.77 (0.48-1.23)	0.273
Single/widowed	**Reference**		**Reference**	
**Alcohol consumption**				
Regular alcohol	1.49 (0.99-2.26)	0.057	1.50 (0.92-2.46)	0.104
No/infrequent alcohol	**Reference**		**Reference**	
**Smoking status**				
Not current smokers	**Reference**		**Reference**	
Non-daily smokers	1.69 (0.79 – 3.58)		1.22 (0.50-2.97)	
Daily smokers	2.13 (1.32 – 3.43)	0.007	1.63 (0.93-2.87)	0.236

*Age adjusted for sex and sex for age: #Fully Adjusted Hazard Ratios adjusted for age, sex, HIV status, hypertension, diabetes mellitus, BMI, marital status, alcohol use and smoking.

For peer review only - http://bmjopen.bmj.com/site/about/guidelines.xhtml

Discussion

In a rural Ugandan population cohort, we found a graded association between low baseline kidney function and subsequent mortality. Participants with severe kidney impairment (eGFR <45mls/min/1.73m^2) had a more than six-fold risk of dying compared to those with eGFR >90mls/min/1.73m^2. This was despite a low prevalence of obesity, diabetes and regular smoking, all key risk factors for kidney disease in high-income countries.

Our study has a number of strengths. It is large and conducted within a well-established population cohort with robust standardised procedures for detailed measurements of covariates such as kidney function and blood pressure, and creatinine was measured according to recommended standards [13]. There is high participation and retention in the study within the local community and regular reporting of mortality and migration by local study workers leading to limited loss to follow-up [13]. Outward migration by predominantly younger participants leads to a degree of selection bias. However, people with chronic health problems often return home so we anticipate capturing the majority of deaths among the baseline cohort, even among those who previously migrated.

However, there were also limitations. This cohort covers a region that is mostly rural, although this is in keeping with the vast majority of people living in Uganda and in many regions of sub-Saharan Africa. Information such as smoking, diabetes and blood pressure were not measured in *both* the 2011 and 2014 surveys of the GPC so our complete case analysis led to reduction in power for the fully-adjusted model. However, there are no substantial differences in characteristics between those with complete and incomplete covariate information, and we see little difference in the effect estimates between age-sex adjusted and fully-adjusted models. We used baseline measures of covariates and were not able to update health related confounders over time. There is likely to be inaccuracy in measurement of kidney function which we categorised using estimated GFR calculated from serum creatinine, a breakdown product of muscle and related to muscle mass and dietary meat intake [16]. Therefore, the prevalence of low BMI in this population where food scarcity is common [19] means that we may have underestimated the prevalence of impaired GFR in this population. In

For peer review only - http://bmjopen.bmj.com/site/about/guidelines.xhtml

addition, validation of GFR estimating equations is currently limited in sub-Saharan Africa [8]. We measured kidney function only once while two measures 3 months apart are required to confirm a diagnosis of chronic kidney disease, and we do not have measures of proteinuria, an important early marker of kidney damage (and predictor of mortality), which may have led to misclassification of some participants [17]. Migration and subsequent loss to follow-up among younger, healthier participants may have led to selection bias with overrepresentation of older participants with health problems including impaired kidney function remaining in the cohort. Finally, we do not know the causes of death, and small numbers of people in lower kidney function subgroups led to limited power and imprecise estimates with wide confidence intervals.

Recent community-based studies of associations of kidney disease suggest important differences in prevalence and associations of impaired kidney function between countries in sub-Saharan Africa. Those including participants from urban areas in South Africa and Nairobi found similar risk factors for those well-established in high-income countries: age, hypertension, diabetes, HIV and female sex [20]. However, cohorts in Malawi and Uganda where the populations are younger, often living in rural areas and with low levels of smoking, obesity and diabetes showed low prevalence of impaired renal function and found a protective association with diabetes, possibly due to hyperfiltration [15 21].

The prospective studies that have looked at the association between mortality and kidney disease in sub-Saharan Africa are limited to cohorts of people which are hospital based [22-25], include patients with only ESKD [26] or HIV [27], or study albuminuria alone [28], and are also limited to short-term follow-up. Our study is the first to demonstrate this association in a general population cohort in sub-Saharan Africa and the results are consistent with studies from high-income countries which have shown a consistent, strong and graded association between impaired (as well as very high) kidney function and risk of subsequent mortality [29]. However, the key difference for our study is that the importance of baseline kidney function can be demonstrated despite the co-existent burden of infectious disease mortality and relatively limited health care system.

For peer review only - http://bmjopen.bmj.com/site/about/guidelines.xhtml

While HIV can cause kidney disease [30] we did not find an interaction between HIV and kidney disease, suggesting that low kidney function has the same pattern of association with mortality among people who are HIV negative. Unexpectedly, we did not find an association between hypertension and mortality in our study despite evidence that it is a key driver of disease and outcomes in sub-Saharan Africa and globally [31]. It is possible that repeated screening within this cohort population has led to higher rates of treatment and control than in other populations, and prevalence of hypertension in this region (14.6%) was lower than the estimated national prevalence of 26.4% [32]. However, it is also possible that the causal relationship between hypertension and kidney damage in sub-Saharan Africa has been oversimplified due to lack of kidney function measurement and longitudinal follow-up. Recent discovery of strong genetic risks for kidney disease in African-Americans has suggested that hypertension and kidney dysfunction may develop in part from shared risk factors [33]. Similarly, diabetes is an independent predictor of mortality among patients with kidney disease and is the leading cause of CKD globally [34]. However, in this population with a low prevalence of diabetes it was not associated with the presence of impaired kidney function or mortality.

In conclusion, in this prospective cohort study based in a rural Ugandan community we found that baseline impaired kidney function was associated with mortality, which remained after adjusting for known shared risk factors such as diabetes, hypertension and tobacco. This suggests that kidney function plays a key role in overall health status in sub-Saharan Africa and, since options for treatment of kidney failure are very restricted, should be included within public health targets. Improved understanding of the determinants of kidney disease and its progression are needed in order to inform interventions for prevention and treatment.

For peer review only - http://bmjopen.bmj.com/site/about/guidelines.xhtml

Acknowledgement

We thank Rose Nabwato, Josephine Kagina Nabukenya, Vincent Arumadri, Francis Namugera, Eva Nambi Ssejjemba and Grace Godfrey Sseremba for the role they played in the study.

Author Contributions Statement

RK conceived the idea. RK, KT, JS and RN and CHH advised on methodology. JS, RN, LS and LAT obtained the funding. RK, KT, BS, RM, PH, MKM, JS and RN collected and curated the data and administered the project. RK and IS analysed the data. RK and LAT wrote the original draft and all authors contributed to and approved the final manuscript.

Additional Information

Collaborators Statement

IS and KT have verified the underlying data. All authors confirm that they had full access to all the data in the study and accept responsibility to submit for publication.

Declaration of interests

The authors declare that there are no conflicts of interest

Data sharing

Owing to data protection concerns, there are restrictions on access to the underlying data. The GPC database contains 25 years of longitudinal data sets on demographics and disease surveillance. All data (census, survey and laboratory) generated through the cohort are stored and curated at the MRC/UVRI and the LSHTM Research Unit. Data access for specific research purposes is possible and has been granted previously. For any data access inquiries, you may contact the director, MRC/UVRI and the LSHTM Research Unit or by email to mrc@mrcuganda.org or the corresponding author.

Funding

This work was funded by GlaxoSmithKline Africa Non-Communicable Disease Open Lab grant number: 8111 as part of a broader multicenter collaborative study between South Africa, Uganda, Malawi and the London School of Hygiene and Tropical Medicine which is collectively identified as the African Research in Kidney Disease (ARK) Network. This work was also funded by Wellcome Trust intermediate clinical fellowship grant number 101143/Z/13/Z awarded to LAT. The General population cohort is funded by the UK Medical Research Council (MRC) and the UK Department for International Development (DFID) under the MRC/DFID Concordat agreement and is also part of the EDCTP2 programme supported by the European Union grant number not applicable. This work was

For peer review only - http://bmjopen.bmj.com/site/about/guidelines.xhtml

also funded by THRiVE-2, a DELTAS Africa grant number DEL-15-011 from Wellcome Trust grant number 107742/Z/15/Z and the UK government which supported JS.

Figure Legends

Figure 1 Title: Study Flow Chart

Figure 2 Title: Hazard ratios for the fully adjusted associations of baseline eGFR and mortality in Uganda

Legend: eGFR- estimated glomerular filtration rate

References

1. Stanifer JW, Jing B, Tolan S, et al. The epidemiology of chronic kidney disease in sub-Saharan Africa: a systematic review and meta-analysis. *Lancet Glob Health* 2014;2(3):e174-81. doi: 10.1016/S2214-109X(14)70002-6 [published Online First: 2014/08/12]
2. Collaboration GBDCKD. Global, regional, and national burden of chronic kidney disease, 1990-2017: a systematic analysis for the Global Burden of Disease Study 2017. *Lancet* 2020;395(10225):709-33. doi: 10.1016/S0140-6736(20)30045-3 [published Online First: 2020/02/18]
3. Wang HE, Gamboa C, Warnock DG, et al. Chronic kidney disease and risk of death from infection. *Am J Nephrol* 2011;34(4):330-6. doi: 10.1159/000330673 [published Online First: 2011/08/24]
4. Sarnak MJ, Levey AS, Schoolwerth AC, et al. Kidney disease as a risk factor for development of cardiovascular disease: a statement from the American Heart Association Councils on Kidney in Cardiovascular Disease, High Blood Pressure Research, Clinical Cardiology, and Epidemiology and Prevention. *Hypertension* 2003;42(5):1050-65. doi: 10.1161/01.HYP.0000102971.85504.7c [published Online First: 2003/11/08]
5. Bukabau JB, Sumaili EK, Cavalier E, et al. Performance of glomerular filtration rate estimation equations in Congolese healthy adults: The inopportunity of the ethnic correction. *PLoS One* 2018;13(3):e0193384. doi: 10.1371/journal.pone.0193384 [published Online First: 2018/03/03]
6. Liyanage T, Ninomiya T, Jha V, et al. Worldwide access to treatment for end-stage kidney disease: a systematic review. *Lancet* 2015;385(9981):1975-82. doi: 10.1016/S0140-6736(14)61601-9 [published Online First: 2015/03/18]
7. Kalyesubula R, Mutyaba I, Rabin T, et al. Trends of admissions and case fatality rates among medical in-patients at a tertiary hospital in Uganda; A four-year retrospective study. *PLoS One* 2019;14(5):e0216060. doi: 10.1371/journal.pone.0216060 [published Online First: 2019/05/16]
8. Fabian J, George JA, Etheredge HR, et al. Methods and reporting of kidney function: a systematic review of studies from sub-Saharan Africa. *Clin Kidney J* 2019;12(6):778-87. doi: 10.1093/ckj/sfz089 [published Online First: 2019/12/07]
9. Delanaye P, Ebert N, Pottel H. Newer GFR Estimating Equations Require Validation in Different Populations. *Am J Kidney Dis* 2017;70(4):586. doi: 10.1053/j.ajkd.2017.06.016 [published Online First: 2017/08/16]

For peer review only - http://bmjopen.bmj.com/site/about/guidelines.xhtml

10. Delanaye P, Mariat C, Maillard N, et al. Are the creatinine-based equations accurate to estimate glomerular filtration rate in African American populations? *Clin J Am Soc Nephrol* 2011;6(4):906-12. doi: 10.2215/CJN.10931210 [published Online First: 2011/03/29]
11. Levey AS, Stevens LA, Schmid CH, et al. A new equation to estimate glomerular filtration rate. *Ann Intern Med* 2009;150(9):604-12. doi: 10.7326/0003-4819-150-9-200905050-00006 [published Online First: 2009/05/06]
12. Nations U. World Bank staff estimates based on the United Nations Population Division's World Urbanization Prospects: 2018 Revision. United Nations. New York: United Nations, 2019:https://population.un.org/wup/Publications/Files/WUP2018-Report.pdf.
13. Asiki G, Murphy G, Nakiyingi-Miiro J, et al. The general population cohort in rural south-western Uganda: a platform for communicable and non-communicable disease studies. *Int J Epidemiol* 2013;42(1):129-41. doi: 10.1093/ije/dys234 [published Online First: 2013/02/01]
14. Seeley J, Kabunga E, Ssembatya J, et al. Understanding kidney disease in rural central Uganda - Findings from a qualitative study. *Glob Public Health* 2020;15(10):1566-77. doi: 10.1080/17441692.2020.1758186 [published Online First: 2020/05/01]
15. Kalyesubula R, Hau JP, Asiki G, et al. Impaired renal function in a rural Ugandan population cohort. *Wellcome Open Res* 2018;3:149. doi: 10.12688/wellcomeopenres.14863.3 [published Online First: 2019/06/25]
16. Myers GL, Miller WG, Coresh J, et al. Recommendations for improving serum creatinine measurement: a report from the Laboratory Working Group of the National Kidney Disease Education Program. *Clin Chem* 2006;52(1):5-18. doi: 10.1373/clinchem.2005.0525144 [published Online First: 2005/12/08]
17. National Kidney F. K/DOQI clinical practice guidelines for chronic kidney disease: evaluation, classification, and stratification. *Am J Kidney Dis* 2002;39(2 Suppl 1):S1-266. [published Online First: 2002/03/21]
18. Murray CJ, Lopez AD, Feehan DM, et al. Validation of the symptom pattern method for analyzing verbal autopsy data. *PLoS Med* 2007;4(11):e327. doi: 10.1371/journal.pmed.0040327 [published Online First: 2007/11/23]
19. Frelat R, Lopez-Ridaura S, Giller KE, et al. Drivers of household food availability in sub-Saharan Africa based on big data from small farms. *Proc Natl Acad Sci U S A* 2016;113(2):458-63. doi: 10.1073/pnas.1518384112 [published Online First: 2015/12/30]
20. George JA, Brandenburg JT, Fabian J, et al. Kidney damage and associated risk factors in rural and urban sub-Saharan Africa (AWI-Gen): a cross-sectional population study. *Lancet Glob Health* 2019;7(12):e1632-e43. doi: 10.1016/S2214-109X(19)30443-7 [published Online First: 2019/11/12]
21. Nakanga WP, Prynn JE, Banda L, et al. Prevalence of impaired renal function among rural and urban populations: findings of a cross-sectional study in Malawi. *Wellcome Open Res* 2019;4:92. doi: 10.12688/wellcomeopenres.15255.1 [published Online First: 2019/10/28]
22. Conroy AL, Opoka RO, Bangirana P, et al. Acute kidney injury is associated with impaired cognition and chronic kidney disease in a prospective cohort of children with severe malaria. *BMC Med* 2019;17(1):98. doi: 10.1186/s12916-019-1332-7 [published Online First: 2019/05/22]
23. Dlamini TAL, Heering PJ, Chivese T, et al. A prospective study of the demographics, management and outcome of patients with acute kidney injury in Cape Town, South Africa. *PLoS One* 2017;12(6):e0177460. doi: 10.1371/journal.pone.0177460 [published Online First: 2017/06/02]
24. Bagasha P, Nakwagala F, Kwizera A, et al. Acute kidney injury among adult patients with sepsis in a low-income country: clinical patterns and short-term outcomes. *BMC Nephrol* 2015;16:4. doi: 10.1186/1471-2369-16-4 [published Online First: 2015/01/17]

For peer review only - http://bmjopen.bmj.com/site/about/guidelines.xhtml

25. Olowu WA, Niang A, Osafo C, et al. Outcomes of acute kidney injury in children and adults in sub-Saharan Africa: a systematic review. *Lancet Glob Health* 2016;4(4):e242-50. doi: 10.1016/S2214-109X(15)00322-8 [published Online First: 2016/03/26]
26. Ashuntantang G, Osafo C, Olowu WA, et al. Outcomes in adults and children with end-stage kidney disease requiring dialysis in sub-Saharan Africa: a systematic review. *Lancet Glob Health* 2017;5(4):e408-e17. doi: 10.1016/S2214-109X(17)30057-8 [published Online First: 2017/02/24]
27. Mulenga LB, Kruse G, Lakhi S, et al. Baseline renal insufficiency and risk of death among HIV-infected adults on antiretroviral therapy in Lusaka, Zambia. *AIDS* 2008;22(14):1821-7. doi: 10.1097/QAD.0b013e328307a051 [published Online First: 2008/08/30]
28. Schutte R, Schmieder RE, Huisman HW, et al. Urinary albumin excretion from spot urine samples predict all-cause and stroke mortality in Africans. *Am J Hypertens* 2014;27(6):811-8. doi: 10.1093/ajh/hpt288 [published Online First: 2014/02/12]
29. Tonelli M, Wiebe N, Culleton B, et al. Chronic kidney disease and mortality risk: a systematic review. *J Am Soc Nephrol* 2006;17(7):2034-47. doi: 10.1681/ASN.2005101085 [published Online First: 2006/06/02]
30. Swanepoel CR, Atta MG, D'Agati VD, et al. Kidney disease in the setting of HIV infection: conclusions from a Kidney Disease: Improving Global Outcomes (KDIGO) Controversies Conference. *Kidney Int* 2018;93(3):545-59. doi: 10.1016/j.kint.2017.11.007 [published Online First: 2018/02/06]
31. Plange-Rhule J, Phillips R, Acheampong JW, et al. Hypertension and renal failure in Kumasi, Ghana. *J Hum Hypertens* 1999;13(1):37-40. doi: 10.1038/sj.jhh.1000726 [published Online First: 1999/02/03]
32. Guwatudde D, Mutungi G, Wesonga R, et al. The Epidemiology of Hypertension in Uganda: Findings from the National Non-Communicable Diseases Risk Factor Survey. *PLoS One* 2015;10(9):e0138991. doi: 10.1371/journal.pone.0138991 [published Online First: 2015/09/26]
33. Nadkarni GN, Coca SG. APOL1 and blood pressure changes in young adults. *Kidney Int* 2017;92(4):793-95. doi: 10.1016/j.kint.2017.05.030 [published Online First: 2017/09/25]
34. Alicic RZ, Rooney MT, Tuttle KR. Diabetic Kidney Disease: Challenges, Progress, and Possibilities. *Clin J Am Soc Nephrol* 2017;12(12):2032-45. doi: 10.2215/CJN.11491116 [published Online First: 2017/05/20]
35. Kalyesubula R, Lunyera J, Makanga G, et al. A 4-year survey of the spectrum of renal disease at a National Referral Hospital Outpatient Clinic in Uganda. *Kidney Int* 2015;87(3):663. doi: 10.1038/ki.2014.411 [published Online First: 2015/02/28]

35

For peer review only - http://bmjopen.bmj.com/site/about/guidelines.xhtml

Study flow diagram

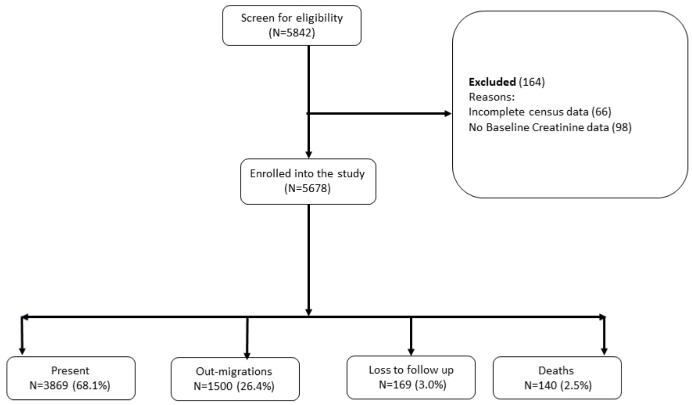

338x190mm (96 x 96 DPI)

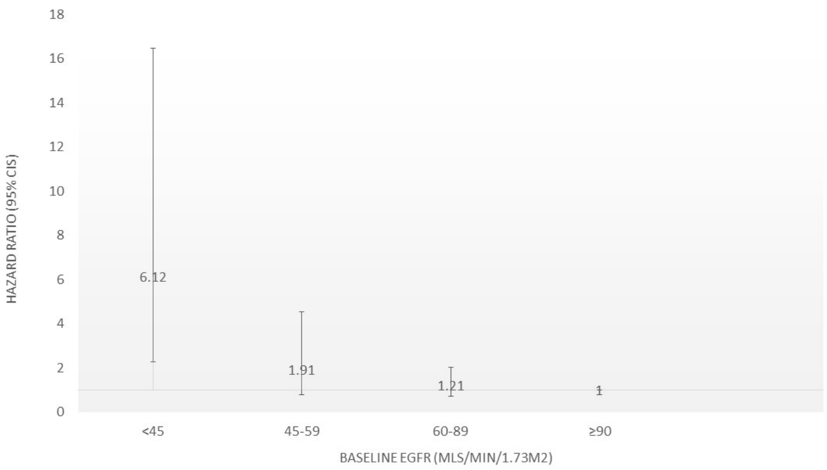

338x190mm (96 x 96 DPI)

Supplementary Appendix for "Association of impaired kidney function with mortality in rural Uganda: results of a general population cohort study"

For peer review only

Supplementary Table 1. Comparison between participants in the main analysis and all enrolled participants.

Variable	Participants with complete data included in main model N=3102	All enrolled participants N=5,678
**eGFR (mls/min/1.73m^2)**		
≥90	2231 (71·9)	4563 (80·3)
60-89	792 (25·6)	1022 (18·0)
45-59	63 (2·0)	73 (1·3)
<45	16 (0·5)	20 (0·4)
**Gender**		
Male	1150 (37·1)	2229 (39·3)
Female	1952 (62·9)	3449 (60·7)
**Age (years), median (IQR)**	45 (35-57)	36 (24-50)
**Age groups, N (%)**		
<35	733 (23·6)	2613 (46·0)
35-44	772 (24·9)	1128 (19·9)
45-54	682 (22·0)	842 (14·9)
55-64	451 (14·5)	540 (9·5)
65-75	291 (9·4)	343 (6·0)
>75	173 (5·6)	212 (3·7)
**Currently married, *N(%)**		
Married	978 (31·5)	1347 (23·7)
Single/widowed	2124 (68·5)	3072 (54·1)
Missing	··	1259 (22·2)
**HIV status, **N (%)**		
Negative	2756 (88·8)	5118 (90·3)
Positive	346 (11·2)	551 (9·7)
Missing	··	9 (0·2)
**Alcohol Consumption, *** N (%)**		
Regular alcohol	1601 (51·6)	2337 (41.1·)
Infrequent/no alcohol	1501 (48·4)	1661 (29.3·)
Missing	··	1680 (29·6)
**Smoking Status, **** N (%)**		
Not current smokers	2757 (88·9)	3617 (63·8)
Non-daily smokers	79 (2·6)	96 (1·6)
Daily smokers	266 (8·5)	284 (5·0)
Missing	··	1681 (29·6)
**Socioeconomic status#**		
Lower	855 (27·5)	1377 (24·2)
Middle	815 (26·3)	1346 (23·7)
Upper	766 (26·7)	1326 (23.4·)
Missing	666 (21·5)	1629 (28·7)
**Hypertension, ## N (%)**		
Normal	2583 (83·3)	3414 (85·4)
Hypertensive	519 (16·7)	584 (14·6)
Missing	··	1680 (29·6)
**Diabetes mellitus, ### N (%)**		
No	3025 (97·5)	3896 (68.·6)
Yes	77 (2·5)	85 (1·.4)
Missing	··	1702 (30·0)
**BMI classification, ####### (kg/m^2) N (%)**		
Underweight (<18·5)	415 (13·4)	665 (11·7)
Normal weight (18·5–24·99)	2029 (65·4)	3879 (68.3·2)
Overweight (25·0–29·99)	484 (15·6)	734 (13·0)
Obese (>30·0)	174 (5·6)	247 (4·3)
Missing	··	153 (2·7)

For peer review only

Supplementary Table 2. Baseline characteristics of participants by five-level categories of kidney function in the general population cohort (n=5,678)

	Estimated glomerular filtration rate (mls/min/1.73m^2)					
Variable	**≥120**	**90-119**	**60-89**	**45-59**	**<45**	**Total**
**Sex**						
Male	709 (36·1)	1144 (44·1)	343 (33·6)	26 (35·6)	7 (35·0)	2229 (39·3)
Female	1257 (63·9)	1453 (56·0)	679 (66·4)	47 (64·4)	13 (65·0)	3449 (60·7)
**Age (years), median (IQR)**	22 (19-28)	41 (33-51)	56 (45-70)	66 (58-76)	68 (42-76)	36 (24-50)
**Age groups, N (%)**						
<35	1784 (90·7)	747 (28·8)	76 (7·4)	3 (4·1)	3 (15·0)	2613 (46·0)
35-44	158 (8·0)	788 (30·3)	176 (17·2)	4 (5·5)	2 (10·0)	1128 (19·9)
45-54	17 (0·9)	602 (23·2)	210 (20·6)	10 (13·7)	3 (15·0)	842 (14·8)
55-64	5 (0·3)	306 (11·8)	214 (20·9)	14 (19·2)	1 (5·0)	540 (9·5)
65-75	2 (0·1)	131 (5·0)	186 (18·2)	19 (26·0)	5 (25·0)	343 (6·0)
>75	0 (0·0)	23 (0·9)	160 (15·7)	23 (31·5)	6 (30·0)	212 (3·7)
**Marital status, N (%)**						
Married	162 (8·2)	691 (26·6)	444 (43·4)	37 (50·7)	13 (65·0)	1347 (23·7)
Single/widowed	888 (45·2)	1608 (61·9)	536 (52·5)	33 (45·2)	7 (35·0)	3072 (54·1)
Missing	916 (46·6)	298 (11·5)	42 (4·1)	3 (4·1)	0 (0·0)	1259 (22·2)
**HIV status, N (%)**						
Negative	1836 (93·4)	2298 (88·5)	901 (88·2)	70 (96·0)	13 (65·0)	5118 (90·1)
Positive	127 (6·5)	297 (11·4)	117 (11·5)	3 (4·1)	7 (35·0)	551 (9·7)
Missing	3 (0·2)	2 (0·1)	4 (0·4)	0 (0·0)	0 (0·0)	9 (0·2)
**Alcohol Consumption, N (%)**						
No	854 (43·4)	1057 (40·7)	393 (38·5)	29 (39·7)	4 (20·0)	2337 (41·2)
Yes	258 (13·1)	906 (34·9)	449 (43·9)	36 (49·3)	12 (60·0)	1661 (29·3)
Missing	854 (43·4)	634 (24·4)	180 (17·6)	8 (11·0)	4 (20·0)	1680 (29·6)
**Smoking Status, N (%)**						
Not current smokers	1061 (54·0)	1740 (67·0)	748 (73·2)	55 (75·3)	13 (65·0)	3617 (63·7)
Non-daily smokers	13 (0·7)	51 (2·0)	30 (2·9)	1 (1·4)	1 (5·0)	96 (1·7)
Daily smokers	38 (1·9)	171 (6·6)	64 (6·3)	9 (12·3)	2 (10·0)	284 (5·0)
Missing	854 (43·4)	635 (24·5)	180 (17·6)	8 (11·0)	4 (20·0)	1681 (29·6)
**Socioeconomic status[#]**						
Lower	417 (21·2)	647 (24·9)	289 (28·3)	21 (28·8)	3 (15·0)	1377 (24·3)
Middle	413 (21·0)	646 (24·9)	257 (25·2)	23 (31·5)	7 (35·0)	1346 (23·7)
Upper	431 (21·9)	598 (23·0)	272 (26·6)	21 (28·8)	4 (20·0)	1326 (23·4)
Missing	705 (35·9)	706 (27·2)	204 (20·0)	8 (11·0)	6 (30·0)	1629 (28·7)
**Hypertension,[##] N (%)**						
Normal	1064 (54·1)	1692 (65·2)	614 (60·1)	35 (47·9)	9 (45·0)	3414 (60·1)
Hypertensive	48 (2·4)	271 (10·4)	228 (22·3)	30 (41·1)	7 (35·0)	584 (10·3)
Missing	854 (43·4)	634 (24·4)	180 (17·6)	8 (11·0)	4 (20·0)	1680 (29·6)
**Diabetes mellitus,[###] N (%)**						
No	1096 (55·8)	1902 (73·2)	815 (79·8)	62 (84·9)	16 (80·0)	3926 (68·5)
Yes	13 (0·7)	49 (1·9)	20 (2·0)	3 (4·1)	0 (0·0)	85 (1·5)
Missing	857 (43·6)	646 (24·9)	187(18·3)	8 (11·0)	4 (20·0)	1702 (30·0)
**BMI classification,[#####] (kg/m^2) N (%)**						
Underweight (<18·5)	185 (9·4)	319 (12·3)	143 (14·0)	14 (19·2)	4 (20·0)	665 (11·7)
Normal weight (18·5–24·99)	1429 (72·7)	1772 (68·2)	623 (61·0)	41 (56·2)	14 (70·0)	3879 (68·3)
Overweight (25·0–29·99)	195 (9·9)	357 (13·8)	166 (16·2)	14 (19·2)	2 (10·0)	734 (12·9)
Obese (≥30·0)	46 (2·3)	121 (4·7)	77 (7·6)	3 (4·1)	0 (0·0)	247 (4·4)
Missing	111 (5·7)	28 (1·1)	13 (1·3)	1 (1·4)	0 (0)	153 (2·7)

**Missing data largely due to different variables being collected in the 2011 and 2014 surveys of the GPC: Socio-economic status (SES) derived from conducting Principle Component Analysis (PCA) using variables relating to household infrastructure and property ownership [##]Hypertension was defined as having a systolic BP ≥90 mmHg, diastolic BP ≥140 mmHg. [###]Diabetes mellitus defined as HBA1C >6.5%, previously diagnosed with diabetes mellitus or being on treatment for diabetes. [####]Body Mass Index (BMI) Classification according to WHO (weight/height^2: kg/m^2).*

For peer review only

Supplementary Table 3. Results of age-sex and fully-adjusted regression models for the association between five category kidney function and mortality in the general population cohort

	Age and sex adjusted		Fully adjusted#	
	**HR (95% CI)**	**P-value**	**HR (95% CI)**	**P-value**
**eGFR (mls/min/1.73m²)**				
≥120	2·68 (1·47-4·87)		1·65 (0·61-4·44)	
90-119	**reference**		**reference**	
60-89	1·14 (0·73-1·77)		1·23 (0·73-2·06)	
45-59	1·67 (0·77-3·60)		1·90 (0·79-4·52)	
<45	5·58 (2·36-13·24)	<0·001	6·06 (2·25-16·37)	0·005
**Age (years)***	1·06 (1·05-1·07)	<0·001	1·06 (1·04-1·08)	<0·001
**Sex***				
Male	**reference**		**reference**	
Female	0·50 (0·36-0·70)	<0·001	0·71 (0·42-1·19)	0·19
**HIV status**				
Positive	2·29 (1·44-3·65)	<0·001	1·69 (0·89-3·23)	0·11
Negative				
**Hypertension**				
Normotensive	**reference**		**reference**	
Hypertensive	0·93 (0·60-1·43)	0·73	0·82 (0·51-1·33)	0·42
**Diabetes mellitus**				
No	**reference**		**reference**	
Yes	0·85 (0·27-2·67)	0·78	1·11 (0·34-3·56)	0·87
**BMI classification**				
Under weight	2·14 (1·49-3·09)		1·70 (1·09-2·66)	
Normal weight	**reference**		**reference**	
Over weight	0·21 (0·07-0·66)		0·20 (0·05-0·84)	
Obese	0·21 (0·03-1·50)	<0·001	0·35 (0·05-2·58)	0·006
**Marital status**				
Married	0·55 (0·37-0·81)	0·003	0·77 (0·48-1·22)	0·27
Single/widowed	**reference**		**reference**	
**Alcohol consumption**				
Regular alcohol	1·49 (0·99-2·26)	0·057	1·50 (0·92-2·45)	0·11
Infrequent/no alcohol	**reference**		**reference**	
**Tobacco use**				
Not current smokers	**reference**		**reference**	
Non-daily smokers	1·69 (0·79-3·58)		1·23 (0·51 - 3·00)	
Daily smokers	2·13 (1·32-3·43)	0·007	1·63 (0·92 - 2·86)	0·24

*Age adjusted for sex and sex for age: # Fully Adjusted Hazard Ratios adjusted for age, sex, HIV status, hypertension, diabetes mellitus, BMI, marital status, alcohol use and smoking. Number of people in the fully adjusted model is 3,102.

For peer review only

STROBE Statement—Checklist of items that should be included in reports of ***cohort studies***

	Item No	Recommendation	Page No
**Title and abstract**	1	(*a*) Indicate the study's design with a commonly used term in the title or the abstract	2
		(*b*) Provide in the abstract an informative and balanced summary of what was done and what was found	2
**Introduction**			
Background/rationale	2	Explain the scientific background and rationale for the investigation being reported	3
Objectives	3	State specific objectives, including any prespecified hypotheses	4
**Methods**			
Study design	4	Present key elements of study design early in the paper	4
Setting	5	Describe the setting, locations, and relevant dates, including periods of recruitment, exposure, follow-up, and data collection	4,5
Participants	6	(*a*) Give the eligibility criteria, and the sources and methods of selection of participants. Describe methods of follow-up	5
		(*b*) For matched studies, give matching criteria and number of exposed and unexposed	
Variables	7	Clearly define all outcomes, exposures, predictors, potential confounders, and effect modifiers. Give diagnostic criteria, if applicable	6
Data sources/ measurement	8*	For each variable of interest, give sources of data and details of methods of assessment (measurement). Describe comparability of assessment methods if there is more than one group	6
Bias	9	Describe any efforts to address potential sources of bias	7
Study size	10	Explain how the study size was arrived at	6
Quantitative variables	11	Explain how quantitative variables were handled in the analyses. If applicable, describe which groupings were chosen and why	6,7
Statistical methods	12	(*a*) Describe all statistical methods, including those used to control for confounding	6,7
		(*b*) Describe any methods used to examine subgroups and interactions	
		(*c*) Explain how missing data were addressed	
		(*d*) If applicable, explain how loss to follow-up was addressed	
		(*e*) Describe any sensitivity analyses	
**Results**			
Participants	13*	(a) Report numbers of individuals at each stage of study—eg numbers potentially eligible, examined for eligibility, confirmed eligible, included in the study, completing follow-up, and analysed	8
		(b) Give reasons for non-participation at each stage	8
		(c) Consider use of a flow diagram	8
Descriptive data	14*	(a) Give characteristics of study participants (eg demographic, clinical, social) and information on exposures and potential confounders	9
		(b) Indicate number of participants with missing data for each variable of interest	9
		(c) Summarise follow-up time (eg, average and total amount)	8,10
Outcome data	15*	Report numbers of outcome events or summary measures over time	8,10

For peer review only

Main results	16	(*a*) Give unadjusted estimates and, if applicable, confounder-adjusted estimates and their precision (eg, 95% confidence interval). Make clear which confounders were adjusted for and why they were included	10,11
		(*b*) Report category boundaries when continuous variables were categorized	
		(*c*) If relevant, consider translating estimates of relative risk into absolute risk for a meaningful time period	8,10
Other analyses	17	Report other analyses done—eg analyses of subgroups and interactions, and sensitivity analyses	10
**Discussion**			
Key results	18	Summarise key results with reference to study objectives	12
Limitations	19	Discuss limitations of the study, taking into account sources of potential bias or imprecision. Discuss both direction and magnitude of any potential bias	12
Interpretation	20	Give a cautious overall interpretation of results considering objectives, limitations, multiplicity of analyses, results from similar studies, and other relevant evidence	12,13,14
Generalisability	21	Discuss the generalisability (external validity) of the study results	14
**Other information**			
Funding	22	Give the source of funding and the role of the funders for the present study and, if applicable, for the original study on which the present article is based	15

*Give information separately for exposed and unexposed groups.

Note: An Explanation and Elaboration article discusses each checklist item and gives methodological background and published examples of transparent reporting. The STROBE checklist is best used in conjunction with this article (freely available on the Web sites of PLoS Medicine at http://www.plosmedicine.org/, Annals of Internal Medicine at http://www.annals.org/, and Epidemiology at http://www.epidem.com/). Information on the STROBE Initiative is available at http://www.strobe-statement.org.

For peer review only

For peer review only - http://bmjopen.bmj.com/site/about/guidelines.xhtml

BMJ Open

Association of impaired kidney function with mortality in rural Uganda: results of a general population cohort study

Journal:	*BMJ Open*
Manuscript ID	bmjopen-2021-051267.R1
Article Type:	Original research
Date Submitted by the Author:	26-Oct-2021
Complete List of Authors:	Kalyesubula, Robert; London School of Hygiene and Tropical Medicine Faculty of Epidemiology and Population Health; Makerere University College of Health Sciences, Physiology and Internal Medicine<br>Sekitoleko, Isaac; MRC/UVRI and LSHTM Research Unit<br>Tomlin, Keith; London School of Hygiene and Tropical Medicine Faculty of Epidemiology and Population Health<br>Hansen, Christian; London School of Hygiene & Tropical Medicine, Infectious Diseases Epidemiology<br>Ssebunya, Billy; MRC/UVRI and LSHTM Research Unit<br>Makanga, Ronald; MRC/UVRI and LSHTM Research Unit<br>Mbonye, Moses Kwizera; MRC/UVRI and LSHTM Research Unit<br>Seeley, Janet; London School of Hygiene and Tropical Medicine, Department of Global Health &Development<br>Smeeth, Liam; London School of Hygiene and Tropical Medicine Faculty of Epidemiology and Public Health<br>Newton, Robert; University of York Department of Health Sciences; Medical Research Council/Uganda Virus Research Institute<br>Tomlinson, Laurie; London School of Hygiene & Tropical Medicine, Epidemiology and Population Health
**Primary Subject Heading**:	Renal medicine
Secondary Subject Heading:	Epidemiology
Keywords:	Nephrology < INTERNAL MEDICINE, PUBLIC HEALTH, EPIDEMIOLOGY

SCHOLARONE™
Manuscripts

BMJ

I, the Submitting Author has the right to grant and does grant on behalf of all authors of the Work (as defined in the below author licence), an exclusive licence and/or a non-exclusive licence for contributions from authors who are: i) UK Crown employees; ii) where BMJ has agreed a CC-BY licence shall apply, and/or iii) in accordance with the terms applicable for US Federal Government officers or employees acting as part of their official duties; on a worldwide, perpetual, irrevocable, royalty-free basis to BMJ Publishing Group Ltd ("BMJ") its licensees and where the relevant Journal is co-owned by BMJ to the co-owners of the Journal, to publish the Work in this journal and any other BMJ products and to exploit all rights, as set out in our licence.

The Submitting Author accepts and understands that any supply made under these terms is made by BMJ to the Submitting Author unless you are acting as an employee on behalf of your employer or a postgraduate student of an affiliated institution which is paying any applicable article publishing charge ("APC") for Open Access articles. Where the Submitting Author wishes to make the Work available on an Open Access basis (and intends to pay the relevant APC), the terms of reuse of such Open Access shall be governed by a Creative Commons licence – details of these licences and which Creative Commons licence will apply to this Work are set out in our licence referred to above.

Other than as permitted in any relevant BMJ Author's Self Archiving Policies, I confirm this Work has not been accepted for publication elsewhere, is not being considered for publication elsewhere and does not duplicate material already published. I confirm all authors consent to publication of this Work and authorise the granting of this licence.

For peer review only

Association of impaired kidney function with mortality in rural Uganda: results of a general population cohort study

Robert Kalyesubula[#1,2 3], Isaac Sekitoleko[1,3], Keith Tomlin[1], Christian Hansen[1,2], Billy Ssebunya[3], Ronald Makanga[3], Moses Kwizera Mbonye[3], Janet Seeley[1,3], Liam Smeeth[1], Robert Newton[3,4], Laurie A. Tomlinson[1]

Affiliations

1. London School of Hygiene and Tropical Medicine, London, UK
2. Makerere University College of Health Sciences, Kampala, Uganda
3. MRC/UVRI and LSHTM Research Unit, Entebbe, Uganda
4. University of York, York, UK

[#]Corresponding author

Dr Robert Kalyesubula

MRC/UVRI & LSHTM Research Unit, Entebbe, Uganda

Email: rkalyesubula@gmail.com

Word Count: 3980 words

For peer review only - http://bmjopen.bmj.com/site/about/guidelines.xhtml

Abstract

Objectives To determine the association between baseline kidney function and subsequent all-cause mortality.

Design and settings A general population-based cohort (GPC) study from rural Uganda.

Participants. 5,678 participants aged 18 years and above (3102 included in the fully adjusted model) with baseline estimated glomerular filtration rate (eGFR) recruited between 2011 and 2015 and followed up to March 2019.

Primary and secondary outcome measures. The associations between baseline eGFR and all-cause mortality was determined using multivariable cox regression.

Results The median age of the participants at baseline was 36 years (IQR 24-50), 60.7% were female, 14.6% hypertensive, 9.7% HIV-positive and 1.8% diabetic. We registered 140 deaths with a median follow-up of 5.0 (IQR 3.7-6.0) years. Adjusting for age and sex, HIV, hypertension, diabetes, BMI, marital status, and alcohol and tobacco use, participants with eGFR ≤45 mls/min/1.73m^2 had six-fold higher mortality compared to those with eGFR ≥90mls/min/1·73m^2 (HR 6·12 (95% CI 2.27-16.45)) with strong evidence of a linear trend for risk of mortality as kidney function declined (P<0.001).

Conclusion In a prospective cohort with high rates of follow-up we found that baseline moderate kidney dysfunction was associated with increased mortality. Improved understanding of the determinants of kidney disease and its progression are needed in order to inform interventions for prevention and treatment.

For peer review only

Strengths and limitations of this study

- This is a large well-established population cohort with robust standardised procedures for detailed measurements of covariates such as kidney function and blood pressure, and creatinine was measured according to recommended international standards.
- We had a high participation and retention in the study within the local community and regular reporting of mortality and migration leading to limited loss to follow-up.
- To our knowledge this is the first time that a long-term prospective association between baseline impaired kidney function and mortality in an adult general population of this size has been demonstrated in sub-Saharan Africa.
- Outward migration by predominantly younger participants and use of a single measurement of creatinine without microalbuminuria may have led to a degree of bias.
- Information such as smoking, diabetes and blood pressure were not measured in both the 2011 and 2014 surveys of the GPC so our complete case analysis led to reduction in power for the fully-adjusted model.

For peer review only - http://bmjopen.bmj.com/site/about/guidelines.xhtml

Introduction

Chronic kidney disease (CKD) affects approximately one in every ten adults across the world and is strongly associated with morbidity and mortality [1 2]. The leading causes of death among people with CKD include cardiovascular disease and infections while a proportion progress to end-stage kidney disease (ESKD) [3 4]. In high-income countries, ESKD is a chronic disease that can be managed with dialysis or kidney transplantation[5]. For low-income countries such as Uganda there is very limited access to kidney replacement therapies and most patients with ESKD die prematurely [6 7]. Compounding the challenge of managing kidney disease in sub-Saharan Africa (SSA) is that population prevalence estimates are limited [1], biochemical methods of measuring kidney function have been suboptimal [8], and the appropriate way to estimate kidney function is uncertain, since equations developed in high-income countries have not been validated for SSA [8-11]. In order to determine the value of developing health services to measure and manage kidney disease, we need to understand the relative importance of kidney function in determining prognosis compared to other chronic diseases in this setting. At present no population-based studies have examined the association between kidney function and mortality in sub-Saharan Africa. Therefore, we sought to determine the association between baseline kidney function and all-cause mortality in a large, well-described general population cohort of people in rural Uganda from 2011-2019.

Methods

Study design and setting

This was a prospective general population cohort (GPC) of people in Kyamulibwa, a rural community 132 kilometres from Kampala, the capital of Uganda. The GPC was established in 1989 by the Medical Research Council (MRC) UK and the Uganda Virus Research Institute (UVRI) to study the epidemiology of HIV infection. Subsequently, other diseases including non-communicable diseases have been examined in the same cohort. The population consists of rural subsistence farmers with a

For peer review only - http://bmjopen.bmj.com/site/about/guidelines.xhtml

few peri-urban dwellers and is similar to the broader rural populations of Uganda which constitute about 76% of the country[12]. This is an open cohort with new births, migrations (inward and outward) and deaths recorded regularly by community volunteers through the annual census. The participant response rate is high, at 95% [13]. Once every two years a survey is conducted to determine diseases and outcomes of interest. We use consecutive sampling for all eligible participants and research hubs are set up in homesteads of community members where the questionnaires are administered and the samples are collected. In 2011-12 and 2014-15, the major foci were diabetes, hypertension, obesity and chronic kidney disease. These diseases were selected because they are among the common non-communicable diseases that have been found to cause high morbidity and mortality. Our participant response rate at baseline was high, at 97.2%, we included 5678 participants out of the available 5842 participants enrolled from census conducted in 2011-12 and 2014-15. We collected data on subsequent mortality for participants who had a baseline creatinine measured in either 2011-2012 or 2014-2015.

Patient and Public Involvement in Research

This research was carried out with participation from members of the public of Kyamulibwa. As a research centre we have a community advisory board (CAB) which gets involved from inception of the studies to their implementation. The community was consulted on the priorities of research for the area in 2012 and non-communicable disease were identified as one of the key areas of research. Through seminars and face to face meetings community members were involved in the development of the key questions and outcome measures of the study on kidney disease. We developed simple tools on how the kidneys work and conducted several awareness campaigns in the villages to explain and get more input from the participants and other members of the community. We also undertook a qualitative study to understand the way community members appreciated kidney disease[14] . Plans to disseminate our work involve sharing results with the community of Kyamulibwa as well as presentation to both national and international forum.

For peer review only - http://bmjopen.bmj.com/site/about/guidelines.xhtml

Participants

We identified potential participants above 18 years who had been recruited consecutively from household visits to a common homesteads called research hubs in the community for creatinine analysis [15]. All participants had given informed consent for specimen storage and future use of their stored samples.

Variables

We extracted baseline demographic characteristics including age, sex, tribe, home address, socioeconomic status and maximum level of education. We also collected information on smoking, alcohol use, exercise patterns and diet in the census rounds of 2011-12 *or* 2014-15 (thus this information was not available for all participants for both rounds). We used participants' height and weight to determine their body mass index (BMI) using weight (kg) /height^2 (m^2). Blood pressure was measured using a digital sphygmomanometer (Omron M4-1). The participant had to be in a sitting position and the mean of the second and third readings taken at 5-minute intervals was used for analysis. Participants with systolic blood pressure ≥140mmHg and/or diastolic blood pressures ≥90mmHg and those who were on treatment for hypertension were classified as hypertensive.

We also analysed blood for creatinine levels, hepatitis B, hepatitis C, HIV-infection and cholesterol levels. We tested venous blood for haemoglobin level using CT -5 Coulter Ac. T 5diff AL [Beckman Coulter, North America]. We performed HIV testing using an approved national algorithm[16] We tested for Hepatitis B surface antigen, Hepatitis C antibody and creatinine level using a Cobas e 601 Auto Analyzer (Roche Diagnostics, North America). Diabetes mellitus was defined as having HbA1C >6.5%, being previously diagnosed with diabetes, or being on current treatment for diabetes. Detailed descriptions of these measurements have been published elsewhere [15]. Creatinine was measured using the enzymatic method traceable to an isotope dilution mass spectrometry method and 93.5% of all participants had a valid creatinine result (6.5% had no blood samples taken, usually

For peer review only - http://bmjopen.bmj.com/site/about/guidelines.xhtml

because consent for this was declined)[15 17]. We calculated eGFR based on the CKD-Epi-creatinine equation without adjustment for ethnicity [18] and classified impaired kidney function in categories analogous to those used to define CKD stages[19]. We collected data on mortality through registers updated monthly through reports from community health workers (verified by verbal autopsy conducted in accordance with standard guidelines by WHO [20]) and cross-checked via annual census.

Statistical Analysis

We conducted a complete-case analysis with regard to baseline creatinine measurements and also excluded participants with missing data regarding outcome or with probable linkage errors. We described the population characteristics according to pre-defined categories of eGFR. We used multivariable Cox modelling to determine the Hazard Ratio (HR) for mortality for each category of eGFR. We tested the proportional hazards assumption using log-minus-log survival plots and visual inspection of Kaplan-Meier curves. Due to missing data for some covariates (such as smoking and alcohol use) which were not collected in *both* the 2011 and 2014 surveys, we present models adjusted for age and sex, and then additionally for baseline HIV status, hypertension, diabetes mellitus, BMI, smoking, alcohol and marital status. Confounders such as diabetes mellitus, hypertension and HIV were included in the analysis model based on previous research showing their associationwith low eGFR in this population[15 21] and after investigation of co-linearity and data sparsity. We present the rate of mortality per 1,000 person-years, and report HR and 95% confidence intervals (CIs).

Because HIV is common in this population and has been associated with increased risk of both kidney impairment and mortality, we examined potential interaction between eGFR and HIV status in the association with mortality by fitting an interaction term in the final adjusted model. In additional analysis, to examine for a 'J-shaped curve' in the association between eGFR and mortality we repeated the main analysis after re-categorisation of eGFR with those ≥120mls/min/1.73m^2 as a separate group. We also analysed for the association between kidney function as continuous eGFR and

For peer review only - http://bmjopen.bmj.com/site/about/guidelines.xhtml

mortality. We analysed all data using STATA version 15.0 (StataCorp, College Station, TX, USA) statistical software.

Ethics

All study participants gave written informed consent to participate in the study. The study was approved by Uganda Virus Research Institute Research and Ethics Committee (UVRI-REC-#HS 1978), the Uganda National Council for Science and Technology (UNCST-#SS 4283) and the London School of Hygiene and Tropical Medicine Observational / Interventions Research Ethics Committee (LSHTM Ethics #21802)

Role of the funding source

The funders had no role in study design, data collection and analysis, decision to publish or preparation of the manuscript.

Results

We included 5,678 participants with kidney function and mortality data available for the analysis (**Figure 1**). The median age of the participants was 36 years (interquartile range (IQR) 24-50 years) with most being female (60.7%) and of normal weight (70.2%) while 9.7% were HIV-positive. The majority (90.5%) of the participants were non-smokers and 58.5% did not consume alcohol. 14.6% were hypertensive and 1.8% of the participants were diabetic (**Table 1**). Comparison of characteristics with differences in baseline characteristics between those with complete and incomplete covariate information are shown in **Supplementary Table 1**. We also included the baseline characteristics of participants by five-level stages of kidney function including eGFR > 120mls/min/1.73m^2 (**Supplementary Table 2**).

During follow-up there were 140 deaths with a median follow-up of 5.0 years (IQR 3.7-6.0). The overall incidence rate of death for the participants enrolled in the study was 4 deaths per 1000 person-years at risk (PYAR). For participants with eGFR >90mls/min/1.73m^2 the incident rate was 3 deaths per

For peer review only - http://bmjopen.bmj.com/site/about/guidelines.xhtml

1000PYARand for those <60mls/min/1.73m^2 it was 27 deaths per 1000 PYAR. During follow-up, 26.4% (1,500/5,678) of participants migrated and 3.0% (169/5,678) were lost to follow-up.

For peer review only

For peer review only - http://bmjopen.bmj.com/site/about/guidelines.xhtml

Table 1. Baseline characteristics of participants categorised by level of kidney function in the general population cohort (N=5,678)

Variable	Estimated glomerular filtration rate (mls/min/1·73m²)				
	**≥90**	**60-89**	**45-59**	**<45**	**Total**
	4563 (80.4)	1022 (18.0)	73 (1.3)	20 (0.4)	
**Sex**					
Male	1853 (40·6)	343 (33·6)	26 (35·6)	7 (35·0)	2229 (39·3)
Female	2710 (59·4)	679 (66·4)	47 (64·4)	13 (65·0)	3449 (60·7)
**Age (years), median (IQR)**	32 (22-44)	56 (45-70)	66 (58-76)	68 (42-76)	36 (24-50)
**Age groups, N (%)**					
<35	2531 (55·5)	76 (7·4)	3 (4·1)	3 (15·0)	2613 (46·0)
35-44	946 (20·7)	176 (17·2)	4 (5·5)	2 (10·0)	1128 (19·9)
45-54	619 (13·6)	210 (20·6)	10 (13·7)	3 (15·0)	842 (14·8)
55-64	311 (6·8)	214 (20·9)	14 (19·2)	1 (5·0)	540 (9·5)
65-75	133 (2·9)	186 (18·2)	19 (26·0)	5 (25·0)	343 (6·0)
>75	23 (0·5)	160 (15·7)	23 (31·5)	6 (30·0)	212 (3·7)
**Marital status, N (%)**					
Single/widowed	853 (18·7)	444 (43·4)	37 (50·7)	13 (65·0)	1347 (23·7)
Married	2496 (54·7)	536 (52·5)	33 (45·2)	7 (35·0)	3072 (54·1)
Missing	1214 (26·6)	42 (4·1)	3 (4·1)	0 (0·0)	1259 (22·2)
**HIV status, N (%)**					
Negative	4134 (90·6)	901 (88·2)	70 (95·9)	13 (65·0)	5118 (90·1)
Positive	424 (9·3)	117 (11·5)	3 (4·1)	7 (35·0)	551 (9·7)
Missing	5 (0·1)	4 (0·4)	0 (0·0)	0 (0·0)	9 (0·2)
**Alcohol Consumption, N (%)**					
Infrequent/no alcohol	1911 (41·9)	393 (38·5)	29 (39·7)	4 (20·0)	2337 (41·2)
Regular alcohol	1164 (25·5)	449 (43·9)	36 (49·3)	12 (60·0)	1661 (29·3)
Missing	1488 (32·6)	180 (17·6)	8 (11·0)	4 (20·0)	1680 (29·6)
**Smoking Status, N (%)**					
Not current smokers	2801 (61·4)	748 (73·2)	55 (75·3)	13 (65·0)	3617 (63·7)
Non-daily smokers	64 (1·4)	30 (2·9)	1 (1·4)	1 (5·0)	96 (1·7)
Daily smokers	209 (4·6)	64 (6·3)	9 (12·3)	2 (10·0)	284 (5·0)
Missing	1489 (32·6)	180 (17·6)	8 (11·0)	4 (20·0)	1681 (29·6)
**Socioeconomic status**					
Lower	1064 (23·3)	289 (28·3)	21 (28·8)	3 (15·0)	1377 (24·3)
Middle	1059 (23·5)	257 (25·2)	23 (31·5)	7 (35·0)	1346 (23·7)
Upper	1029 (22·6)	272 (26·6)	21 (28·8)	4 (20·0)	1326 (23·4)
Missing	1411 (30·9)	204 (20·0)	8 (11·0)	6 (30·0)	1629 (28·7)
**Hypertension, N (%)**					
Normal	2756 (60·4)	614 (60·1)	35 (48·0)	9 (45·0)	3414 (60·1)
Hypertensive	319 (7·0)	228 (22·3)	30 (41·1)	7 (35·0)	584 (10·3)
Missing	1488 (32·6)	180 (17·6)	8 (11·0)	4 (20·0)	1680 (29·6)
**Diabetes mellitus, N (%)**					
No	2998 (65·7)	815 (79·8)	62 (84·9)	16 (100·0)	3891 (68·5)
Yes	62 (1·4)	20 (2·0)	3 (4·1)	0 (0·0)	85 (1·5)
Missing	1503 (32·9)	187 (18·3)	8 (11·0)	4 (20·0)	1702 (30·0)
**BMI classification, (kg/m²) N (%)**					
Underweight (<18·5)	504 (11·1)	143 (14·0)	14 (19·2)	4 (10·0)	665 (11·7)
Normal weight (18·5–24·99)	3201 (70·2)	623 (61·0)	41 (56·2)	14 (70·0)	3879 (68·3)
Overweight (25·0–29·99)	552 (12·1)	166 (16·2)	14 (19·2)	2 (10·0)	734 (12·9)
Obese (>30·0)	167 (3·7)	77 (7·5)	3 (4·1)	0 (0·0)	247 (4·4)
Missing	139 (3·1)	13 (1·3)	1 (1·4)	0 (0·0)	153 (2·7)

Missing data mainly arises due to different variables being collected in the 2011 and 2014 surveys of the GPC·

For peer review only

Age-sex and fully adjusted associations with mortality are shown in **Table 2** and **Figure 2**. In age and sex adjusted analyses, mortality was associated with poorer kidney function, being HIV positive, being unmarried or widowed, low BMI and regular smoking. In the fully-adjusted model, low kidney function remained associated with increased risk of mortality, (HR 6.12, 95% CI 2.27-16.45) for those with eGFR <45mls/min/1.73m^2 compared to those with eGFR ≥90mls/min/1.73m^2. The test for trend showed strong evidence (P<0.001) that the mortality rate increased progressively as category of baseline kidney function decreased. On analysis for the association between kidney function as continuous eGFR and mortality, there was very strong evidence (P<0.001) of a non-linear association between eGFR and mortality largely re-enforcing the U-shaped relationship. (**Supplementary Table 3**).

There was no evidence of an interaction between HIV and kidney function with risk of mortality in the fully adjusted model (P=0.672). In an additional analysis, when high eGFR was included as a separate category there was weak evidence of a 'J-shaped curve' (**Supplementary Table 4**). In age-sex adjusted analysis, baseline eGFR ≥120mls/min/1.73m^2 was associated with increased risk of mortality (HR 2.68, 95% CI 1.47-4.87) compared to the reference category of 90-119mls/min/1.73m^2 while in fully-adjusted analyses confidence intervals crossed the null (HR 1.65, 95% CI 0.61-4.44).

For peer review only - http://bmjopen.bmj.com/site/about/guidelines.xhtml

For peer review only

Table 2. Results of age-sex and fully-adjusted regression models for the association between kidney function and mortality in the general population cohort

		Age and sex adjusted		Fully adjusted[#](n=3102)	
	Number of participants	HR (95% CI)	P-value	HR (95% CI)	P-value
**eGFR (mls/min/1.73m²)**	5678				
≥90		**Reference**		**Reference**	
60-89		1·11 (0·73-1·71)		1·21 (0·73-2·03)	
45-59		1·72 (0·81-3·69)		1·91 (0·80-4·54)	
<45		5·97 (2·55-13·98)	<0·001	6·12 (2·27-16·45)	0·003
**Age (years)***		1·06 (1·05-1·07)	<0·001	1·05 (1·03-1·07)	<0·001
**Sex***					
Male		**Reference**		**Reference**	
Female		0·50 (0·36-0·70)	<0·001	0·70 (0·42-1·07)	0·188
**HIV status**	5669				
Positive		2·29 (1·43-3·65)	<0·001	1·67 (0·87-3·18)	0·120
Negative		**Reference**		**Reference**	
**Hypertension**	3998				
Normotensive		**Reference**		**Reference**	
Hypertensive		0·93 (0·60-1·43)	0·725	1·08 (0·58-2·01)	0·433
**Diabetes mellitus**	3976				
No		**Reference**		**Reference**	
Yes		0·85 (0·27-2·67)	0·777	1·11 (0·35-3·57)	0·861
**BMI classification**	5525				
Under weight		2·14 (1·49-3·09)		1·71 (1·10-2·67)	
Normal weight		**Reference**		**Reference**	
Over weight		0·21 (0·07-0·66)		0·20 (0·05-0·84)	
Obese		0·21 (0·03-1·50)	<0·001	0·35 (0·48-2·55)	0·006
**Marital status**	4419				
Currently married		0·55 (0·37-0·81)	0·003	0·77 (0·48-1·23)	0·273
Single/widowed		**Reference**		**Reference**	
**Alcohol consumption**	3998				
Regular alcohol		1·49 (0·99-2·26)	0.057	1·50 (0·92-2·46)	0·104
No/infrequent alcohol		**Reference**		**Reference**	
**Smoking status**	3997				
Not current smokers		**Reference**		**Reference**	
Non-daily smokers		1·69 (0·79 – 3·58)		1·22 (0·50-2·97)	
Daily smokers		2·13 (1·32 – 3·43)	0·007	1·63 (0·93-2·87)	0·236

*Age adjusted for sex and sex for age: [#]Fully Adjusted Hazard Ratios adjusted for age, sex, HIV status, hypertension, diabetes mellitus, BMI, marital status, alcohol use and smoking.

For peer review only

Discussion

In a rural Ugandan population cohort, we found a graded association between low baseline kidney function and subsequent mortality. Participants with severe kidney impairment (eGFR <45mls/min/1.73m^2) had a more than six-fold risk of dying compared to those with eGFR >90mls/min/1.73m^2. This was despite a low prevalence of obesity, diabetes and regular smoking, all key risk factors for kidney disease in high-income countries.

Our study has a number of strengths. It is large and conducted within a well-established population cohort with robust standardised procedures for detailed measurements of covariates such as blood pressure, and creatinine was measured according to recommended standards [13]. There is high participation and retention in the study within the local community and regular reporting of mortality and migration by local study workers leading to limited loss to follow-up [13]. Outward migration by predominantly younger participants leads to a degree of selection bias. However, people with chronic health problems often return home so we anticipate capturing the majority of deaths among the baseline cohort, even among those who previously migrated.

However, there were also limitations. This cohort covers a region that is mostly rural, although this is in keeping with the vast majority of people living in Uganda and in many regions of sub-Saharan Africa. Information such as smoking, diabetes and blood pressure were not measured in *both* the 2011 and 2014 surveys of the GPC so our complete case analysis led to reduction in power for the fully-adjusted model. However, there are no substantial differences in baseline characteristics between those with complete and incomplete covariate information, and we see little difference in the effect estimates between age-sex adjusted and fully-adjusted models. We used baseline measures of covariates and were not able to update health related confounders over time. There is likely to be inaccuracy in measurement of kidney function which we categorised using estimated GFR calculated from serum creatinine, a breakdown product of muscle and related to muscle mass and dietary meat intake [17]. Therefore, the prevalence of low BMI in this population where food scarcity is common [22] means that we may have underestimated the prevalence of impaired GFR in

For peer review only - http://bmjopen.bmj.com/site/about/guidelines.xhtml

this population. In addition, validation of GFR estimating equations is currently limited in sub-Saharan Africa [8]. We measured kidney function only once while two measures 3 months apart are required to confirm a diagnosis of chronic kidney disease, and we do not have measures of proteinuria, an important early marker of kidney damage (and predictor of mortality), which may have led to misclassification of some participants[19]. Migration and subsequent loss to follow-up among younger, healthier participants may have led to selection bias with overrepresentation of older participants with health problems including impaired kidney function remaining in the cohort. Finally, we do not know the causes of death, and small numbers of people in lower kidney function subgroups led to limited power and imprecise estimates with wide confidence intervals.

Recent community-based studies of associations of kidney disease suggest important differences in prevalence and associations of impaired kidney function between countries in sub-Saharan Africa. Those including participants from urban areas in South Africa and Nairobi found similar risk factors for those well-established in high-income countries: age, hypertension, diabetes, HIV and female sex [23]. However, cohorts in Malawi and Uganda where the populations are younger, often living in rural areas and with low levels of smoking, obesity and diabetes showed low prevalence of impaired kidney function and found an association with diabetes, possibly due to hyperfiltration [15 24].

The prospective studies that have looked at the association between mortality and kidney disease in sub-Saharan Africa are limited to cohorts of people which are hospital based [25-28], include patients with only ESKD [29] or HIV [30], or study albuminuria alone [31], and are also limited to short-term follow-up. Our study is the first to demonstrate this association in a general population cohort in sub-Saharan Africa and the results are consistent with studies from high-income countries which have shown a consistent, strong and graded association between impaired (as well as very high) kidney function and risk of subsequent mortality [32]. However, the key difference for our study is that the importance of baseline kidney function can be demonstrated despite the co-existent burden of infectious disease mortality and relatively limited health care system.

For peer review only - http://bmjopen.bmj.com/site/about/guidelines.xhtml

For peer review only

While HIV can cause kidney disease [33] we did not find an interaction between HIV and kidney disease, suggesting that low kidney function has the same pattern of association with mortality among people who are HIV negative. Unexpectedly, we did not find an association between hypertension and mortality in our study despite evidence that it is a key driver of disease and outcomes in sub-Saharan Africa and globally [34]. It is possible that repeated screening within this cohort population has led to higher rates of treatment and control than in other populations, and prevalence of hypertension in this region (14.6%) was lower than the estimated national prevalence of 26.4% [35]. However, it is also possible that the causal relationship between hypertension and kidney damage in sub-Saharan Africa has been oversimplified due to lack of kidney function measurement and longitudinal follow-up. Recent discovery of strong genetic risks for kidney disease in African-Americans has suggested that hypertension and kidney dysfunction may develop in part from shared risk factors [36]. Similarly, diabetes is an independent predictor of mortality among patients with kidney disease and is the leading cause of CKD globally [37]. However, in this population with a low prevalence of diabetes it was not associated with the presence of impaired kidney function or mortality.

In conclusion, in this prospective cohort study based in a rural Ugandan community we found that baseline impaired kidney function was associated with mortality, which remained after adjusting for known shared risk factors such as diabetes, hypertension and tobacco. This suggests that kidney function plays a key role in overall health status in sub-Saharan Africa and, since options for treatment of kidney failure are very restricted, should be included within public health targets. Improved understanding of the determinants of kidney disease and its progression are needed in order to inform interventions for prevention and treatment.

For peer review only - http://bmjopen.bmj.com/site/about/guidelines.xhtml

Acknowledgement

We thank Rose Nabwato, Josephine Kagina Nabukenya, Vincent Arumadri, Francis Namugera, Eva Nambi Ssejjemba and Grace Godfrey Sseremba for the role they played in the study.

Author Contributions Statement

RK conceived the idea. RK, KT, JS and RN and CHH advised on methodology. JS, RN, LS and LAT obtained the funding. RK, KT, BS, RM, MKM, JS and RN collected and curated the data and administered the project. RK and IS analysed the data. RK and LAT wrote the original draft and all authors contributed to and approved the final manuscript.

Additional Information

Collaborators Statement

IS and KT have verified the underlying data. All authors confirm that they had full access to all the data in the study and accept responsibility to submit for publication.

Declaration of interests

The authors declare that there are no conflicts of interest

Data sharing

Owing to data protection concerns, there are restrictions on access to the underlying data. The GPC database contains 25 years of longitudinal data sets on demographics and disease surveillance. All data (census, survey and laboratory) generated through the cohort are stored and curated at the MRC/UVRI and the LSHTM Research Unit. Data access for specific research purposes is possible and has been granted previously. For any data access inquiries, you may contact the director, MRC/UVRI and the LSHTM Research Unit or by email to mrc@mrcuganda.org or the corresponding author.

Funding

This work was funded by GlaxoSmithKline Africa Non-Communicable Disease Open Lab grant number: 8111 as part of a broader multicenter collaborative study between South Africa, Uganda, Malawi and the London School of Hygiene and Tropical Medicine which is collectively identified as the African Research in Kidney Disease (ARK) Network. This work was also funded by Wellcome Trust intermediate clinical fellowship grant number 101143/Z/13/Z awarded to LAT. The General population cohort is funded by the UK Medical Research Council (MRC) and the UK Department for International Development (DFID) under the MRC/DFID Concordat agreement and is also part of the EDCTP2 programme supported by the European Union grant number not applicable. This work was

For peer review only - http://bmjopen.bmj.com/site/about/guidelines.xhtml

For peer review only

also funded by THRiVE-2, a DELTAS Africa grant number DEL-15-011 from Wellcome Trust grant number 107742/Z/15/Z and the UK government which supported JS.

Figure Legends

Figure 1 Title: Study Flow Chart

Figure 2 Title: Hazard ratios for the fully adjusted associations of baseline eGFR and mortality in Uganda

Legend: eGFR- estimated glomerular filtration rate

For peer review only

References

1. Stanifer JW, Jing B, Tolan S, et al. The epidemiology of chronic kidney disease in sub-Saharan Africa: a systematic review and meta-analysis. *Lancet Glob Health* 2014;2(3):e174-81. doi: 10.1016/S2214-109X(14)70002-6 [published Online First: 2014/08/12]
2. Collaboration GBDCKD. Global, regional, and national burden of chronic kidney disease, 1990-2017: a systematic analysis for the Global Burden of Disease Study 2017. *Lancet* 2020;395(10225):709-33. doi: 10.1016/S0140-6736(20)30045-3 [published Online First: 2020/02/18]
3. Wang HE, Gamboa C, Warnock DG, et al. Chronic kidney disease and risk of death from infection. *Am J Nephrol* 2011;34(4):330-6. doi: 10.1159/000330673 [published Online First: 2011/08/24]
4. Sarnak MJ, Levey AS, Schoolwerth AC, et al. Kidney disease as a risk factor for development of cardiovascular disease: a statement from the American Heart Association Councils on Kidney in Cardiovascular Disease, High Blood Pressure Research, Clinical Cardiology, and Epidemiology and Prevention. *Hypertension* 2003;42(5):1050-65. doi: 10.1161/01.HYP.0000102971.85504.7c [published Online First: 2003/11/08]
5. Thurlow JS, Joshi M, Yan G, et al. Global Epidemiology of End-Stage Kidney Disease and Disparities in Kidney Replacement Therapy. *Am J Nephrol* 2021;52(2):98-107. doi: 10.1159/000514550 [published Online First: 2021/03/23]
6. Liyanage T, Ninomiya T, Jha V, et al. Worldwide access to treatment for end-stage kidney disease: a systematic review. *Lancet* 2015;385(9981):1975-82. doi: 10.1016/S0140-6736(14)61601-9 [published Online First: 2015/03/18]
7. Kalyesubula R, Mutyaba I, Rabin T, et al. Trends of admissions and case fatality rates among medical in-patients at a tertiary hospital in Uganda; A four-year retrospective study. *PLoS One* 2019;14(5):e0216060. doi: 10.1371/journal.pone.0216060 [published Online First: 2019/05/16]
8. Fabian J, George JA, Etheredge HR, et al. Methods and reporting of kidney function: a systematic review of studies from sub-Saharan Africa. *Clin Kidney J* 2019;12(6):778-87. doi: 10.1093/ckj/sfz089 [published Online First: 2019/12/07]
9. Delanaye P, Ebert N, Pottel H. Newer GFR Estimating Equations Require Validation in Different Populations. *Am J Kidney Dis* 2017;70(4):586. doi: 10.1053/j.ajkd.2017.06.016 [published Online First: 2017/08/16]

10. Delanaye P, Mariat C, Maillard N, et al. Are the creatinine-based equations accurate to estimate glomerular filtration rate in African American populations? *Clin J Am Soc Nephrol* 2011;6(4):906-12. doi: 10.2215/CJN.10931210 [published Online First: 2011/03/29]
11. Levey AS, Stevens LA, Schmid CH, et al. A new equation to estimate glomerular filtration rate. *Ann Intern Med* 2009;150(9):604-12. doi: 10.7326/0003-4819-150-9-200905050-00006 [published Online First: 2009/05/06]
12. Nations U. World Bank staff estimates based on the United Nations Population Division's World Urbanization Prospects: 2018 Revision. United Nations. New York: United Nations, 2019:https://population.un.org/wup/Publications/Files/WUP2018-Report.pdf.
13. Asiki G, Murphy G, Nakiyingi-Miiro J, et al. The general population cohort in rural south-western Uganda: a platform for communicable and non-communicable disease studies. *Int J Epidemiol* 2013;42(1):129-41. doi: 10.1093/ije/dys234 [published Online First: 2013/02/01]
14. Seeley J, Kabunga E, Ssembatya J, et al. Understanding kidney disease in rural central Uganda - Findings from a qualitative study. *Glob Public Health* 2020;15(10):1566-77. doi: 10.1080/17441692.2020.1758186 [published Online First: 2020/05/01]
15. Kalyesubula R, Hau JP, Asiki G, et al. Impaired renal function in a rural Ugandan population cohort. *Wellcome Open Res* 2018;3:149. doi: 10.12688/wellcomeopenres.14863.3 [published Online First: 2019/06/25]
16. Galiwango RM, Musoke R, Lubyayi L, et al. Evaluation of current rapid HIV test algorithms in Rakai, Uganda. *J Virol Methods* 2013;192(1-2):25-7. doi: 10.1016/j.jviromet.2013.04.003 [published Online First: 2013/04/16]
17. Myers GL, Miller WG, Coresh J, et al. Recommendations for improving serum creatinine measurement: a report from the Laboratory Working Group of the National Kidney Disease Education Program. *Clin Chem* 2006;52(1):5-18. doi: 10.1373/clinchem.2005.0525144 [published Online First: 2005/12/08]
18. Bukabau JB, Sumaili EK, Cavalier E, et al. Performance of glomerular filtration rate estimation equations in Congolese healthy adults: The inopportunity of the ethnic correction. *PLoS One* 2018;13(3):e0193384. doi: 10.1371/journal.pone.0193384 [published Online First: 2018/03/03]
19. Inker LA, Astor BC, Fox CH, et al. KDOQI US commentary on the 2012 KDIGO clinical practice guideline for the evaluation and management of CKD. *Am J Kidney Dis* 2014;63(5):713-35. doi: 10.1053/j.ajkd.2014.01.416 [published Online First: 2014/03/22]
20. Murray CJ, Lopez AD, Feehan DM, et al. Validation of the symptom pattern method for analyzing verbal autopsy data. *PLoS Med* 2007;4(11):e327. doi: 10.1371/journal.pmed.0040327 [published Online First: 2007/11/23]
21. Babua C, Kalyesubula R, Okello E, et al. Cardiovascular risk factors among patients with chronic kidney disease attending a tertiary hospital in Uganda. *Cardiovasc J Afr* 2015;26(4):177-80. doi: 10.5830/CVJA-2015-045 [published Online First: 2015/09/26]
22. Frelat R, Lopez-Ridaura S, Giller KE, et al. Drivers of household food availability in sub-Saharan Africa based on big data from small farms. *Proc Natl Acad Sci U S A* 2016;113(2):458-63. doi: 10.1073/pnas.1518384112 [published Online First: 2015/12/30]
23. George JA, Brandenburg JT, Fabian J, et al. Kidney damage and associated risk factors in rural and urban sub-Saharan Africa (AWI-Gen): a cross-sectional population study. *Lancet Glob Health* 2019;7(12):e1632-e43. doi: 10.1016/S2214-109X(19)30443-7 [published Online First: 2019/11/12]
24. Nakanga WP, Prynn JE, Banda L, et al. Prevalence of impaired renal function among rural and urban populations: findings of a cross-sectional study in Malawi. *Wellcome Open Res* 2019;4:92. doi: 10.12688/wellcomeopenres.15255.1 [published Online First: 2019/10/28]
25. Conroy AL, Opoka RO, Bangirana P, et al. Acute kidney injury is associated with impaired cognition and chronic kidney disease in a prospective cohort of children with severe malaria.

For peer review only - http://bmjopen.bmj.com/site/about/guidelines.xhtml

BMC Med 2019;17(1):98. doi: 10.1186/s12916-019-1332-7 [published Online First: 2019/05/22]

26. Dlamini TAL, Heering PJ, Chivese T, et al. A prospective study of the demographics, management and outcome of patients with acute kidney injury in Cape Town, South Africa. *PLoS One* 2017;12(6):e0177460. doi: 10.1371/journal.pone.0177460 [published Online First: 2017/06/02]
27. Bagasha P, Nakwagala F, Kwizera A, et al. Acute kidney injury among adult patients with sepsis in a low-income country: clinical patterns and short-term outcomes. *BMC Nephrol* 2015;16:4. doi: 10.1186/1471-2369-16-4 [published Online First: 2015/01/17]
28. Olowu WA, Niang A, Osafo C, et al. Outcomes of acute kidney injury in children and adults in sub-Saharan Africa: a systematic review. *Lancet Glob Health* 2016;4(4):e242-50. doi: 10.1016/S2214-109X(15)00322-8 [published Online First: 2016/03/26]
29. Ashuntantang G, Osafo C, Olowu WA, et al. Outcomes in adults and children with end-stage kidney disease requiring dialysis in sub-Saharan Africa: a systematic review. *Lancet Glob Health* 2017;5(4):e408-e17. doi: 10.1016/S2214-109X(17)30057-8 [published Online First: 2017/02/24]
30. Mulenga LB, Kruse G, Lakhi S, et al. Baseline renal insufficiency and risk of death among HIV-infected adults on antiretroviral therapy in Lusaka, Zambia. *AIDS* 2008;22(14):1821-7. doi: 10.1097/QAD.0b013e328307a051 [published Online First: 2008/08/30]
31. Schutte R, Schmieder RE, Huisman HW, et al. Urinary albumin excretion from spot urine samples predict all-cause and stroke mortality in Africans. *Am J Hypertens* 2014;27(6):811-8. doi: 10.1093/ajh/hpt288 [published Online First: 2014/02/12]
32. Tonelli M, Wiebe N, Culleton B, et al. Chronic kidney disease and mortality risk: a systematic review. *J Am Soc Nephrol* 2006;17(7):2034-47. doi: 10.1681/ASN.2005101085 [published Online First: 2006/06/02]
33. Swanepoel CR, Atta MG, D'Agati VD, et al. Kidney disease in the setting of HIV infection: conclusions from a Kidney Disease: Improving Global Outcomes (KDIGO) Controversies Conference. *Kidney Int* 2018;93(3):545-59. doi: 10.1016/j.kint.2017.11.007 [published Online First: 2018/02/06]
34. Plange-Rhule J, Phillips R, Acheampong JW, et al. Hypertension and renal failure in Kumasi, Ghana. *J Hum Hypertens* 1999;13(1):37-40. doi: 10.1038/sj.jhh.1000726 [published Online First: 1999/02/03]
35. Guwatudde D, Mutungi G, Wesonga R, et al. The Epidemiology of Hypertension in Uganda: Findings from the National Non-Communicable Diseases Risk Factor Survey. *PLoS One* 2015;10(9):e0138991. doi: 10.1371/journal.pone.0138991 [published Online First: 2015/09/26]
36. Nadkarni GN, Coca SG. APOL1 and blood pressure changes in young adults. *Kidney Int* 2017;92(4):793-95. doi: 10.1016/j.kint.2017.05.030 [published Online First: 2017/09/25]
37. Alicic RZ, Rooney MT, Tuttle KR. Diabetic Kidney Disease: Challenges, Progress, and Possibilities. *Clin J Am Soc Nephrol* 2017;12(12):2032-45. doi: 10.2215/CJN.11491116 [published Online First: 2017/05/20]

For peer review only - http://bmjopen.bmj.com/site/about/guidelines.xhtml

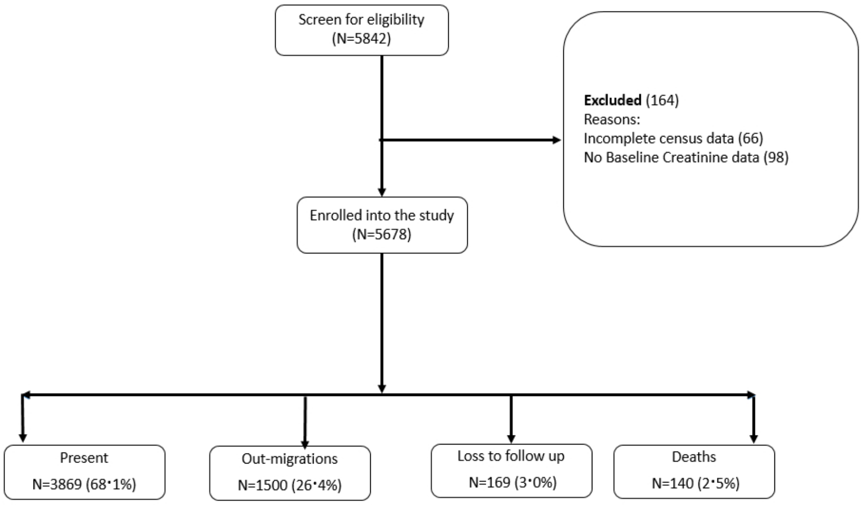

eGFR: estimated glomerural filtration rate.

468x239mm (57 x 57 DPI)

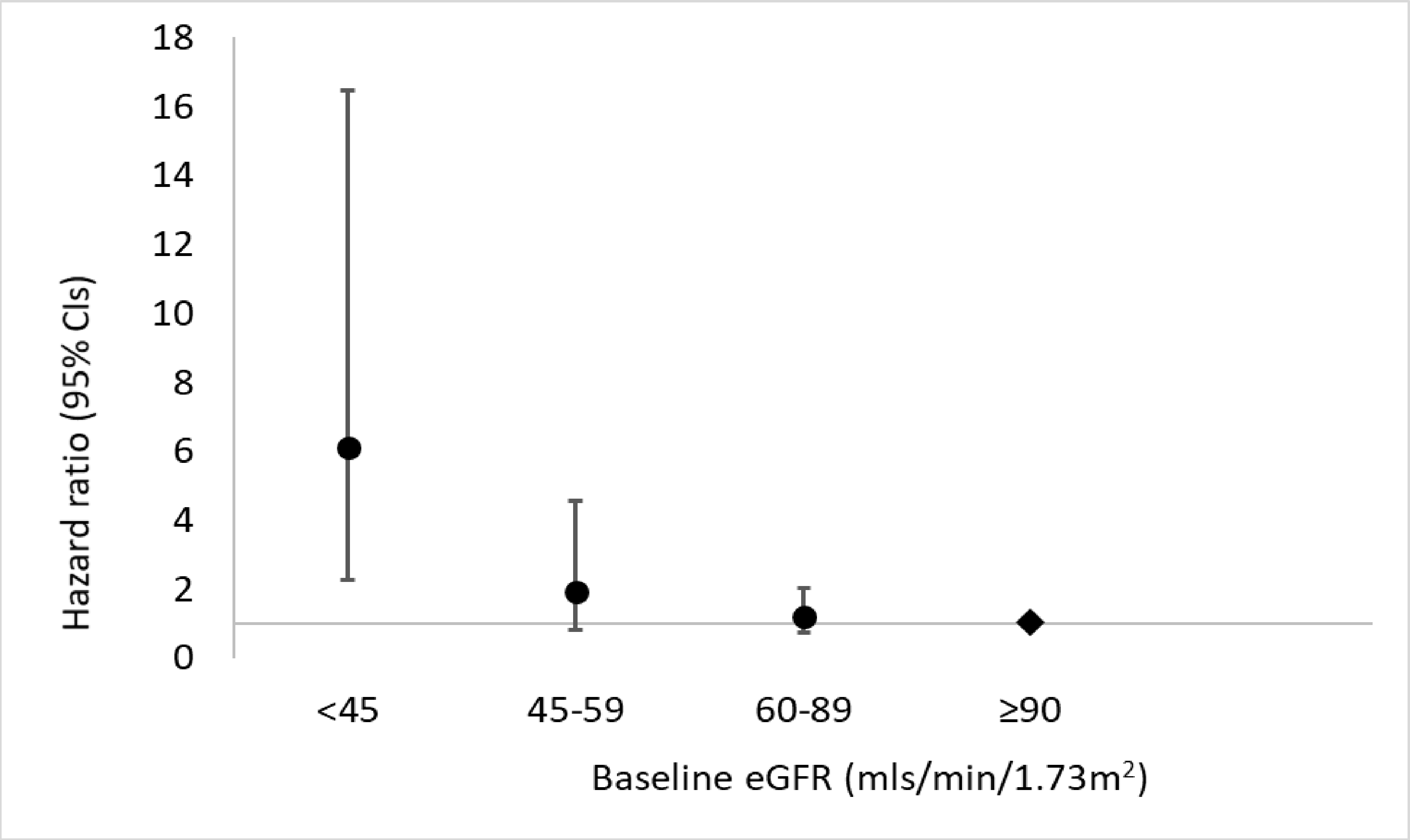
18
16
14
12
10
8
6
4
2
0
Hazard ratio (95% CIs)
<45
45-59
60-89
≥90
Baseline eGFR (mls/min/1.73m2)

Supplementary Appendix for "Association of impaired kidney function with mortality in rural Uganda: results of a general population cohort study"

For peer review only

For peer review only - http://bmjopen.bmj.com/site/about/guidelines.xhtml

Supplementary Table 1. Comparison between participants showing differences in baseline characteristics between those with complete and incomplete covariate information.

Variable	Participants with complete data included in main model N=3102	Participants excluded from the final model N=2567	P-value
**eGFR (mls/min/1.73m^2)**			
≥90	2231 (71·9)	2332 (90.5)	
60-89	792 (25·6)	230 (8.9)	
45-59	63 (2·0)	10 (0.4)	
<45	16 (0·5)	4 (0·2)	<0.001
**Gender**			
Male	1150 (37·1)	1079 (41.9)	
Female	1952 (62·9)	1497 (58.1)	<0.001
**Age (years), median (IQR)**	45 (35-57)	24 (19-36)	<0.001
**Age groups, N (%)**			
<35	733 (23·6)	1880 (73.0)	
35-44	772 (24·9)	356 (13.8)	
45-54	682 (22·0)	160 (6.2)	
55-64	451 (14·5)	89 (3.5)	
65-75	291 (9·4)	52 (2.0)	
>75	173 (5·6)	39 (1.5)	<0.001
**Currently married, *N(%)**			
Married	978 (31·5)	369 (14.3)	
Single/widowed	2124 (68·5)	948 (36.8)	
Missing	··	1259 (48.9)	<0.001
**HIV status, **N (%)**			
Negative	2756 (88·8)	2362 (91.7)	
Positive	346 (11·2)	205 (8.0)	
Missing	··	9 (0·3)	<0.001
**Alcohol Consumption, *** N (%)**			
Regular alcohol	1601 (51·6)	736 (28.6)	
Infrequent/no alcohol	1501 (48·4)	160 (6.2)	
Missing	··	1680 (65.2)	<0.001
**Smoking Status, **** N (%)**			
Not current smokers	2757 (88·9)	860 (33.4)	
Non-daily smokers	79 (2·6)	17 (0.7)	
Daily smokers	266 (8·5)	18 (0.7)	
Missing	··	1681 (65.3)	<0.001
**Socioeconomic status[#]**			
Lower	855 (27·5)	522 (20.3)	
Middle	815 (26·3)	531 (20.6)	
Upper	766 (26·7)	560 (21.7)	
Missing	666 (21·5)	963 (37.4)	<0.001
**Hypertension, [##] N (%)**			
Normal	2583 (83·3)	831 (32.3)	
Hypertensive	519 (16·7)	65 (2.5))	
Missing	··	1680 (65.2)	<0.001
**Diabetes mellitus, ### N (%)**			
No	3025 (97·5)	866 (33.6)	
Yes	77 (2·5)	8 (0.3)	
Missing	··	1702 (66.1)	<0.001
**BMI classification, [#######] (kg/m^2) N (%)**			
Underweight (<18·5)	415 (13·4)	250 (9.7)	
Normal weight (18·5–24·99)	2029 (65·4)	1850 (71.8)	
Overweight (25·0–29·99)	484 (15·6)	250 (9.7)	

For peer review only - http://bmjopen.bmj.com/site/about/guidelines.xhtml

Obese (>30·0)	174 (5·6)	73 (2.8)	
Missing	··	153 (5.9)	<0.001

**Missing data largely due to different variables being collected in the 2011 and 2014 surveys of the GPC: Socio-economic status (SES) derived from conducting Principle Component Analysis (PCA) using variables relating to household infrastructure and property ownership ##Hypertension was defined as having a systolic BP ≥90 mmHg, diastolic BP ≥140 mmHg. ###Diabetes mellitus defined as HBA1C >6.5%, previously diagnosed with diabetes mellitus or being on treatment for diabetes. ####Body Mass Index (BMI) Classification according to WHO (weight/height^2: kg/m^2).*

For peer review only

For peer review only - http://bmjopen.bmj.com/site/about/guidelines.xhtml

Supplementary Table 2. Baseline characteristics of participants by five-level categories of kidney function in the general population cohort (n=5,678)

	Estimated glomerular filtration rate (mls/min/1.73m^2)					
Variable	≥120	90-119	60-89	45-59	<45	Total
**Sex**						
Male	709 (36·1)	1144 (44·1)	343 (33·6)	26 (35·6)	7 (35·0)	2229 (39·3)
Female	1257 (63·9)	1453 (56·0)	679 (66·4)	47 (64·4)	13 (65·0)	3449 (60·7)
**Age (years), median (IQR)**	22 (19-28)	41 (33-51)	56 (45-70)	66 (58-76)	68 (42-76)	36 (24-50)
**Age groups, N (%)**						
<35	1784 (90·7)	747 (28·8)	76 (7·4)	3 (4·1)	3 (15·0)	2613 (46·0)
35-44	158 (8·0)	788 (30·3)	176 (17·2)	4 (5·5)	2 (10·0)	1128 (19·9)
45-54	17 (0·9)	602 (23·2)	210 (20·6)	10 (13·7)	3 (15·0)	842 (14·8)
55-64	5 (0·3)	306 (11·8)	214 (20·9)	14 (19·2)	1 (5·0)	540 (9·5)
65-75	2 (0·1)	131 (5·0)	186 (18·2)	19 (26·0)	5 (25·0)	343 (6·0)
>75	0 (0·0)	23 (0·9)	160 (15·7)	23 (31·5)	6 (30·0)	212 (3·7)
**Marital status, N (%)**						
Married	162 (8·2)	691 (26·6)	444 (43·4)	37 (50·7)	13 (65·0)	1347 (23·7)
Single/widowed	888 (45·2)	1608 (61·9)	536 (52·5)	33 (45·2)	7 (35·0)	3072 (54·1)
Missing	916 (46·6)	298 (11·5)	42 (4·1)	3 (4·1)	0 (0·0)	1259 (22·2)
**HIV status, N (%)**						
Negative	1836 (93·4)	2298 (88·5)	901 (88·2)	70 (96·0)	13 (65·0)	5118 (90·1)
Positive	127 (6·5)	297 (11·4)	117 (11·5)	3 (4·1)	7 (35·0)	551 (9·7)
Missing	3 (0·2)	2 (0·1)	4 (0·4)	0 (0·0)	0 (0·0)	9 (0·2)
**Alcohol Consumption, N (%)**						
No	854 (43·4)	1057 (40·7)	393 (38·5)	29 (39·7)	4 (20·0)	2337 (41·2)
Yes	258 (13·1)	906 (34·9)	449 (43·9)	36 (49·3)	12 (60·0)	1661 (29·3)
Missing	854 (43·4)	634 (24·4)	180 (17·6)	8 (11·0)	4 (20·0)	1680 (29·6)
**Smoking Status, N (%)**						
Not current smokers	1061 (54·0)	1740 (67·0)	748 (73·2)	55 (75·3)	13 (65·0)	3617 (63·7)
Non-daily smokers	13 (0·7)	51 (2·0)	30 (2·9)	1 (1·4)	1 (5·0)	96 (1·7)
Daily smokers	38 (1·9)	171 (6·6)	64 (6·3)	9 (12·3)	2 (10·0)	284 (5·0)
Missing	854 (43·4)	635 (24·5)	180 (17·6)	8 (11·0)	4 (20·0)	1681 (29·6)
**Socioeconomic status**[#]						
Lower	417 (21·2)	647 (24·9)	289 (28·3)	21 (28·8)	3 (15·0)	1377 (24·3)
Middle	413 (21·0)	646 (24·9)	257 (25·2)	23 (31·5)	7 (35·0)	1346 (23·7)
Upper	431 (21·9)	598 (23·0)	272 (26·6)	21 (28·8)	4 (20·0)	1326 (23·4)
Missing	705 (35·9)	706 (27·2)	204 (20·0)	8 (11·0)	6 (30·0)	1629 (28·7)
**Hypertension,** [##] **N (%)**						
Normal	1064 (54·1)	1692 (65·2)	614 (60·1)	35 (47·9)	9 (45·0)	3414 (60·1)
Hypertensive	48 (2·4)	271 (10·4)	228 (22·3)	30 (41·1)	7 (35·0)	584 (10·3)
Missing	854 (43·4)	634 (24·4)	180 (17·6)	8 (11·0)	4 (20·0)	1680 (29·6)
**Diabetes mellitus,** [###] **N (%)**						
No	1096 (55·8)	1902 (73·2)	815 (79·8)	62 (84·9)	16 (80·0)	3926 (68·5)
Yes	13 (0·7)	49 (1·9)	20 (2·0)	3 (4·1)	0 (0·0)	85 (1·5)
Missing	857 (43·6)	646 (24·9)	187(18·3)	8 (11·0)	4 (20·0)	1702 (30·0)
**BMI classification,** [#####] **(kg/m^2) N (%)**						
Underweight (<18·5)	185 (9·4)	319 (12·3)	143 (14·0)	14 (19·2)	4 (20·0)	665 (11·7)
Normal weight (18·5–24·99)	1429 (72·7)	1772 (68·2)	623 (61·0)	41 (56·2)	14 (70·0)	3879 (68·3)
Overweight (25·0–29·99)	195 (9·9)	357 (13·8)	166 (16·2)	14 (19·2)	2 (10·0)	734 (12·9)
Obese (≥30·0)	46 (2·3)	121 (4·7)	77 (7·6)	3 (4·1)	0 (0·0)	247 (4·4)
Missing	111 (5·7)	28 (1·1)	13 (1·3)	1 (1·4)	0 (0)	153 (2·7)

**Missing data largely due to different variables being collected in the 2011 and 2014 surveys of the GPC: Socio-economic status (SES) derived from conducting Principle Component Analysis (PCA) using variables relating to household infrastructure and property ownership [##]Hypertension was defined as having a systolic BP ≥90 mmHg, diastolic BP ≥140 mmHg. [###]Diabetes mellitus defined as HBA1C >6.5%, previously diagnosed with diabetes mellitus or being on treatment for diabetes. [####]Body Mass Index (BMI) Classification according to WHO (weight/height^2: kg/m^2).*

For peer review only - http://bmjopen.bmj.com/site/about/guidelines.xhtml

For peer review only

Supplementary Table 3. Results of age-sex and fully-adjusted regression models for the association between kidney function (continuous eGFR) and mortality in the general population cohort.

	Number of participants	Age and sex adjusted HR (95% CI)	Age and sex adjusted P-value	Fully adjusted#(n=3102) HR (95% CI)	Fully adjusted#(n=3102) P-value
eGFR (mls/min/1.73m²) per SD	5678	0.27 (0.15-0.46)	<0.001	0.23 (0.11-0.51)	<0.001
Squared eGFR (mls/min/1.73m²) Per SD	5678	1.09 (1.01-1.15)	0.026	1.16 (1.04-1.29)	0.009

**Age adjusted for sex and sex for age: #Fully Adjusted Hazard Ratios adjusted for age, sex, HIV status, hypertension, diabetes mellitus, BMI, marital status, alcohol use and smoking. SD=22.02 mls/min/1.73m²*

For peer review only

For peer review only - http://bmjopen.bmj.com/site/about/guidelines.xhtml

Supplementary Table 4. Results of age-sex and fully-adjusted regression models for the association between five category kidney function and mortality in the general population cohort

	Age and sex adjusted		Fully adjusted[#]	
	**HR (95% CI)**	**P-value**	**HR (95% CI)**	**P-value**
**eGFR (mls/min/1.73m^2)**				
≥120	2·68 (1·47-4·87)		1·65 (0·61-4·44)	
90-119	**reference**		**reference**	
60-89	1·14 (0·73-1·77)		1·23 (0·73-2·06)	
45-59	1·67 (0·77-3·60)		1·90 (0·79-4·52)	
<45	5·58 (2·36-13·24)	<0·001	6·06 (2·25-16·37)	0·005
**Age (years)***	1·06 (1·05-1·07)	<0·001	1·06 (1·04-1·08)	<0·001
**Sex***				
Male	**reference**		**reference**	
Female	0·50 (0·36-0·70)	<0·001	0·71 (0·42-1·19)	0·19
**HIV status**				
Positive	2·29 (1·44-3·65)	<0·001	1·69 (0·89-3·23)	0·11
Negative				
**Hypertension**				
Normotensive	**reference**		**reference**	
Hypertensive	0·93 (0·60-1·43)	0·73	0·82 (0·51-1·33)	0·42
**Diabetes mellitus**				
No	**reference**		**reference**	
Yes	0·85 (0·27-2·67)	0·78	1·11 (0·34-3·56)	0·87
**BMI classification**				
Under weight	2·14 (1·49-3·09)		1·70 (1·09-2·66)	
Normal weight	**reference**		**reference**	
Over weight	0·21 (0·07-0·66)		0·20 (0·05-0·84)	
Obese	0·21 (0·03-1·50)	<0·001	0·35 (0·05-2·58)	0·006
**Marital status**				
Married	0·55 (0·37-0·81)	0·003	0·77 (0·48-1·22)	0·27
Single/widowed	**reference**		**reference**	
**Alcohol consumption**				
Regular alcohol	1·49 (0·99-2·26)	0·057	1·50 (0·92-2·45)	0·11
Infrequent/no alcohol	**reference**		**reference**	
**Tobacco use**				
Not current smokers	**reference**		**reference**	
Non-daily smokers	1·69 (0·79-3·58)		1·23 (0·51 - 3·00)	
Daily smokers	2·13 (1·32-3·43)	0·007	1·63 (0·92 - 2·86)	0·24

**Age adjusted for sex and sex for age: [#] Fully Adjusted Hazard Ratios adjusted for age, sex, HIV status, hypertension, diabetes mellitus, BMI, marital status, alcohol use and smoking. Number of people in the fully adjusted model is 3,102.*

For peer review only - http://bmjopen.bmj.com/site/about/guidelines.xhtml

STROBE Statement—Checklist of items that should be included in reports of ***cohort studies***

	Item No	Recommendation	Page No
**Title and abstract**	1	(*a*) Indicate the study's design with a commonly used term in the title or the abstract	1
		(*b*) Provide in the abstract an informative and balanced summary of what was done and what was found	2
**Introduction**			
Background/rationale	2	Explain the scientific background and rationale for the investigation being reported	3
Objectives	3	State specific objectives, including any prespecified hypotheses	4
**Methods**			
Study design	4	Present key elements of study design early in the paper	4
Setting	5	Describe the setting, locations, and relevant dates, including periods of recruitment, exposure, follow-up, and data collection	4,7
Participants	6	(*a*) Give the eligibility criteria, and the sources and methods of selection of participants. Describe methods of follow-up	5
		(*b*) For matched studies, give matching criteria and number of exposed and unexposed	
Variables	7	Clearly define all outcomes, exposures, predictors, potential confounders, and effect modifiers. Give diagnostic criteria, if applicable	6
Data sources/ measurement	8*	For each variable of interest, give sources of data and details of methods of assessment (measurement). Describe comparability of assessment methods if there is more than one group	6
Bias	9	Describe any efforts to address potential sources of bias	7
Study size	10	Explain how the study size was arrived at	6
Quantitative variables	11	Explain how quantitative variables were handled in the analyses. If applicable, describe which groupings were chosen and why	6,7
Statistical methods	12	(*a*) Describe all statistical methods, including those used to control for confounding	6,7
		(*b*) Describe any methods used to examine subgroups and interactions	
		(*c*) Explain how missing data were addressed	
		(*d*) If applicable, explain how loss to follow-up was addressed	
		(*e*) Describe any sensitivity analyses	
**Results**			
Participants	13*	(a) Report numbers of individuals at each stage of study—eg numbers potentially eligible, examined for eligibility, confirmed eligible, included in the study, completing follow-up, and analysed	8
		(b) Give reasons for non-participation at each stage	8
		(c) Consider use of a flow diagram	8
Descriptive data	14*	(a) Give characteristics of study participants (eg demographic, clinical, social) and information on exposures and potential confounders	9
		(b) Indicate number of participants with missing data for each variable of interest	9
		(c) Summarise follow-up time (eg, average and total amount)	8,10
Outcome data	15*	Report numbers of outcome events or summary measures over time	8,10

For peer review only

Main results	16	(*a*) Give unadjusted estimates and, if applicable, confounder-adjusted estimates and their precision (eg, 95% confidence interval). Make clear which confounders were adjusted for and why they were included	10,11
		(*b*) Report category boundaries when continuous variables were categorized	
		(*c*) If relevant, consider translating estimates of relative risk into absolute risk for a meaningful time period	8,10
Other analyses	17	Report other analyses done—eg analyses of subgroups and interactions, and sensitivity analyses	10
**Discussion**			
Key results	18	Summarise key results with reference to study objectives	12
Limitations	19	Discuss limitations of the study, taking into account sources of potential bias or imprecision. Discuss both direction and magnitude of any potential bias	12
Interpretation	20	Give a cautious overall interpretation of results considering objectives, limitations, multiplicity of analyses, results from similar studies, and other relevant evidence	12,13,14
Generalisability	21	Discuss the generalisability (external validity) of the study results	14
**Other information**			
Funding	22	Give the source of funding and the role of the funders for the present study and, if applicable, for the original study on which the present article is based	15

*Give information separately for exposed and unexposed groups.

Note: An Explanation and Elaboration article discusses each checklist item and gives methodological background and published examples of transparent reporting. The STROBE checklist is best used in conjunction with this article (freely available on the Web sites of PLoS Medicine at http://www.plosmedicine.org/, Annals of Internal Medicine at http://www.annals.org/, and Epidemiology at http://www.epidem.com/). Information on the STROBE Initiative is available at http://www.strobe-statement.org.

For peer review only

BMJ Open

Association of impaired kidney function with mortality in rural Uganda: results of a general population cohort study

Journal:	*BMJ Open*
Manuscript ID	bmjopen-2021-051267.R2
Article Type:	Original research
Date Submitted by the Author:	26-Jan-2022
Complete List of Authors:	Kalyesubula, Robert; London School of Hygiene and Tropical Medicine Faculty of Epidemiology and Population Health; Makerere University College of Health Sciences, Physiology and Internal Medicine<br>Sekitoleko, Isaac; MRC/UVRI and LSHTM Research Unit<br>Tomlin, Keith; London School of Hygiene and Tropical Medicine Faculty of Epidemiology and Population Health<br>Hansen, Christian; London School of Hygiene & Tropical Medicine, Infectious Diseases Epidemiology<br>Ssebunya, Billy; MRC/UVRI and LSHTM Research Unit<br>Makanga, Ronald; MRC/UVRI and LSHTM Research Unit<br>Mbonye, Moses Kwizera; MRC/UVRI and LSHTM Research Unit<br>Seeley, Janet; London School of Hygiene and Tropical Medicine, Department of Global Health &Development<br>Smeeth, Liam; London School of Hygiene and Tropical Medicine Faculty of Epidemiology and Public Health<br>Newton, Robert; University of York Department of Health Sciences; Medical Research Council/Uganda Virus Research Institute<br>Tomlinson, Laurie; London School of Hygiene & Tropical Medicine, Epidemiology and Population Health
**Primary Subject Heading**:	Renal medicine
Secondary Subject Heading:	Epidemiology
Keywords:	Nephrology < INTERNAL MEDICINE, PUBLIC HEALTH, EPIDEMIOLOGY

SCHOLARONE™
Manuscripts

I, the Submitting Author has the right to grant and does grant on behalf of all authors of the Work (as defined in the below author licence), an exclusive licence and/or a non-exclusive licence for contributions from authors who are: i) UK Crown employees; ii) where BMJ has agreed a CC-BY licence shall apply, and/or iii) in accordance with the terms applicable for US Federal Government officers or employees acting as part of their official duties; on a worldwide, perpetual, irrevocable, royalty-free basis to BMJ Publishing Group Ltd ("BMJ") its licensees and where the relevant Journal is co-owned by BMJ to the co-owners of the Journal, to publish the Work in this journal and any other BMJ products and to exploit all rights, as set out in our licence.

The Submitting Author accepts and understands that any supply made under these terms is made by BMJ to the Submitting Author unless you are acting as an employee on behalf of your employer or a postgraduate student of an affiliated institution which is paying any applicable article publishing charge ("APC") for Open Access articles. Where the Submitting Author wishes to make the Work available on an Open Access basis (and intends to pay the relevant APC), the terms of reuse of such Open Access shall be governed by a Creative Commons licence – details of these licences and which Creative Commons licence will apply to this Work are set out in our licence referred to above.

Other than as permitted in any relevant BMJ Author's Self Archiving Policies, I confirm this Work has not been accepted for publication elsewhere, is not being considered for publication elsewhere and does not duplicate material already published. I confirm all authors consent to publication of this Work and authorise the granting of this licence.

For peer review only

Association of impaired kidney function with mortality in rural Uganda: results of a general population cohort study

Robert Kalyesubula[#1,2 3], Isaac Sekitoleko[1,3], Keith Tomlin[1], Christian Hansen[1,2], Billy Ssebunya[3], Ronald Makanga[3], Moses Kwizera Mbonye[3], Janet Seeley[1,3], Liam Smeeth[1], Robert Newton[3,4], Laurie A. Tomlinson[1]

Affiliations

1. London School of Hygiene and Tropical Medicine, London, UK
2. Makerere University College of Health Sciences, Kampala, Uganda
3. MRC/UVRI and LSHTM Research Unit, Entebbe, Uganda
4. University of York, York, UK

[#]Corresponding author

Dr Robert Kalyesubula

MRC/UVRI & LSHTM Research Unit, Entebbe, Uganda

Email: rkalyesubula@gmail.com

Word Count: 3903 words

For peer review only - http://bmjopen.bmj.com/site/about/guidelines.xhtml

Abstract

Objectives To determine the association between baseline kidney function and subsequent all-cause mortality.

Design and settings A general population-based cohort (GPC) study from rural Uganda.

Participants People aged 18 years and above with measured baseline estimated glomerular filtration rate (eGFR), recruited in survey rounds in 2011-12 or 2014-15 and followed up to March 2019.

Outcome measure The primary outcome was all-cause mortality, identified through reports from community health workers, verified by verbal autopsy. The association between baseline eGFR category and mortality was determined using multivariable cox regression.

Results Of 5,812 participants in both rounds we included 5,678 (97.7%) participants with kidney function and mortality data: median age was 36 years (IQR 24-50), 60.7% female, 10.3% hypertensive, 9.8% HIV-positive and 1.5% diabetic. During a median follow-up of 5.0 years (IQR 3.7-6.0) there were 140 deaths. In age- and sex-adjusted analyses, eGFR <45ml/min/1.73m^2 at baseline was associated with a 5.97 (95% CI 2.55-13.98) increased risk of mortality compared to those with baseline eGFR >90ml/min/1.73m^2. After inclusion of additional confounders (HIV, BMI, Diabetes, hypertension, smoking, alcohol and smoking status) into the model eGFR <45ml/min/1.73m^2 at baseline remained strongly associated with mortality (HR 6.12, 95% CI 2.27-16.45) although sample size fell to 3,102. Test for trend showed strong evidence (P<0.001) that the mortality rate increased progressively as category of baseline kidney function decreased. When very high eGFR was included as a separate category in age-sex adjusted analysis, baseline eGFR ≥120ml/min/1.73m^2 was associated with increased risk of mortality (HR 2.68, 95% CI 1.47-4.87) compared to the reference category of 90-119ml/min/1.73m^2.

Conclusion In a prospective cohort in rural Uganda we found that impaired baseline kidney function was associated with subsequently increased total mortality. Improved understanding of the determinants of kidney disease and its progression are needed in order to inform interventions for prevention and treatment.

For peer review only - http://bmjopen.bmj.com/site/about/guidelines.xhtml

Strengths and limitations of the study

Strengths.

It is the first data of its kind from sub-Saharan Africa, generated in a large, well-established population cohort with robust standardised procedures for detailed measurements of covariates such as blood pressure, and creatinine was measured according to recommended standards.

There is high participation and retention in the study within the local community and regular reporting of mortality and migration by local study workers leading to limited loss to follow-up.

Limitations

There was missing data for covariates such as smoking, diabetes and blood pressure for about one-third of participants so the complete case analysis led to reduction in power for the fully adjusted model.

Participants in the 2011-12 round, who were more likely to have had complete data for potential confounders, also had longer follow-up time over which they could have died.

We used baseline measures of covariates and were not able to update health related confounders over time.

There is likely to be inaccuracy in measurement of kidney function which we categorised using estimated GFR calculated from serum creatinine, a breakdown product of muscle and related to muscle mass and dietary meat intake.

For peer review only

For peer review only - http://bmjopen.bmj.com/site/about/guidelines.xhtml

Summary Box

What is already known

- Impaired kidney function is independently associated with subsequent all-cause mortality in cohorts from high-income countries.
- It was not known if this was the case in sub-Saharan Africa, where there is a young population, a high burden of infectious disease mortality and low prevalence of many non-communicable diseases.

What this study adds

- This study demonstrates that impaired baseline kidney function is associated with all-cause mortality in a general-population cohort of adults in rural Uganda.
- This suggests that kidney function plays a key role in overall health status in sub-Saharan Africa and should be considered as a public health priority.

For peer review only

For peer review only - http://bmjopen.bmj.com/site/about/guidelines.xhtml

Introduction

Chronic kidney disease (CKD) affects approximately one in every ten adults across the world and is strongly associated with morbidity and mortality.[1 2] The leading causes of death among people with CKD include cardiovascular disease and infections while a proportion progress to end-stage kidney disease (ESKD). [3 4] In high-income countries, ESKD is a chronic disease that can be managed with dialysis or kidney transplantation.[5] However, for low-income countries such as Uganda there is very limited access to kidney replacement therapies and most patients with ESKD die prematurely.[6 7] Compounding the challenge of managing kidney disease in sub-Saharan Africa (SSA) is that population prevalence estimates are limited,[1] biochemical methods of measuring kidney function have been suboptimal,[8] and the appropriate way to estimate kidney function is uncertain, since equations developed in high-income countries have not been validated for SSA.[8-11] In order to determine the value of developing health services to measure and manage kidney disease, we need to understand the relative importance of kidney function in determining prognosis compared to other chronic diseases in this setting. At present no population-based studies have examined the association between kidney function and mortality in sub-Saharan Africa. Therefore, we sought to determine the association between baseline kidney function and all-cause mortality in a large, well-described general population cohort of people in rural Uganda from 2011-2019.

Methods

Study design and setting

This was a prospective general population cohort (GPC) of adults in Kyamulibwa, a rural community 132 kilometres from Kampala, the capital of Uganda. The population consists of rural subsistence farmers with a few peri-urban dwellers and is similar to the broader rural populations of Uganda which constitute about 76% of the country.[12] The GPC was established in 1989 by the Medical Research Council (MRC) UK and the Uganda Virus Research Institute (UVRI) to study the

For peer review only - http://bmjopen.bmj.com/site/about/guidelines.xhtml

epidemiology of HIV infection.[13] Subsequently, other diseases of interest including non-communicable diseases have been examined in biannual rounds in the same cohort. It is an open cohort with new births, migrations (inward and outward) and deaths recorded regularly by community volunteers through the annual census.

For this study we selected a subset of the GPC using information from the 2011-12 and 2014-15 rounds of the GPC in which a major disease of interest was chronic kidney disease and thus baseline information on kidney function was available.[14] The outcome was total mortality among participants who had a baseline creatinine measured in either 2011-2012 or 2014-2015.

Patient and Public Involvement in Research

Research within the GPC is carried out with participation from members of the public of Kyamulibwa who participate in a community advisory board (CAB) which is involved from inception of studies to their implementation. The community is consulted on the priorities of research for the area and through seminars and face-to-face meetings community members are involved in the development of key questions and outcome measures. In relation to kidney disease, we have developed simple tools on how the kidneys work and conducted awareness campaigns in the villages to explain and get input from the participants and other members of the community. We plan to disseminate the results both of this work and that of other kidney-related projects with the community of Kyamulibwa.

Participants

We identified potential participants aged above 18 years who were recruited consecutively from household visits in each village. Questionnaires were administered and samples collected at research hubs set up in homesteads of a community member in each location.

Variables

For peer review only

Each round of the GPC records baseline demographic characteristics including age, sex, tribe and maximum level of education as well as HIV status using an approved national algorithm.[15] We used participants' height and weight to determine their body mass index (BMI) using weight (kg) /height^2 (m^2). We tested for creatinine level using a Cobas e 601 Auto Analyzer (Roche Diagnostics, North America), using the enzymatic method traceable to an isotope dilution mass spectrometry method)[14 16]. We calculated estimated glomerular filtration rate (eGFR) based on the CKD-Epi-creatinine equation without adjustment for ethnicity[17] and classified impaired kidney function in categories analogous to those used to define CKD stages.[18] Detailed descriptions of these measurements have been previously published.[14]

Further to these near complete characteristics, in keeping with the different disease areas investigated in different rounds of the GPC, we also collected information on, marital status, smoking, alcohol use, diabetes and blood pressure. This information was the focus of the 2011-12 round so data collection was near complete but was also collected, though not completely, in 2014-15. Blood pressure was measured using a digital sphygmomanometer (Omron M4-1) after the participant was seated and the mean of the second and third readings taken at 5-minute intervals was used for analysis. Participants with systolic blood pressure ≥140mmHg and/or diastolic blood pressures ≥90mmHg, and those who were on treatment for hypertension, were classified as hypertensive. Diabetes mellitus was defined as having HbA1C >6.5%, being previously diagnosed with diabetes, or being on current treatment for diabetes.

We collected data on mortality through registers updated monthly through reports from community health workers, verified by verbal autopsy (a method used to ascertain the cause of a death based on an interview with next of kin or other caregivers used where no other routine systems are in place and where many people die at home), conducted in accordance with standard guidelines by WHO, and cross-checked via annual census.[19]

For peer review only - http://bmjopen.bmj.com/site/about/guidelines.xhtml

Statistical Analysis

We conducted a complete-case analysis with regard to baseline creatinine measurements and also excluded participants with missing data regarding outcome or with probable linkage errors. We described the population characteristics according to pre-defined categories of eGFR. We present the rate of mortality per 1,000 person-years and use multivariable Cox modelling to determine the Hazard Ratio (HR) and 95% Confidence Intervals (95% CI) for mortality for each category of eGFR. In Cox regression we included participants up to their last point of follow-up, so participants who later migrated contributed follow-up time to the analysis while they were present in the area. Participants included in the baseline round also contributed follow-up time if they returned to the area after a period of out-migration.

We tested the proportional hazards assumption using log-minus-log survival plots and visual inspection of Kaplan-Meier curves. Due to incomplete data for some covariates, we present sequentially adjusted models to demonstrate the effect of additional covariate adjustment on the relationship between eGFR and mortality. Thus, we present models adjusted for age and sex, and then additionally for baseline HIV status, hypertension, diabetes mellitus, BMI, smoking, alcohol and marital status. These potential confounders were defined a priori, based on existing literature showing their association with low eGFR and mortality in this population[14 20] and included after investigation of co-linearity and data sparsity.

Because HIV is common in this population and has been associated with increased risk of both kidney impairment and mortality, we examined potential interaction between baseline eGFR and HIV status in the association with mortality by fitting an interaction term in the final adjusted model.

In additional analysis, to examine for a 'J-shaped curve' in the association between eGFR and mortality we repeated the main analysis after re-categorisation of eGFR with those ≥120ml/min/1.73m^2 as a separate group. We analysed all data using STATA version 15.0 (StataCorp, College Station, TX, USA) statistical software.

Ethics

For peer review only - http://bmjopen.bmj.com/site/about/guidelines.xhtml

All study participants gave written informed consent to participation, specimen storage and future use of their stored samples. The study was approved by Uganda Virus Research Institute Research and Ethics Committee (UVRI-REC-#HS 1978), the Uganda National Council for Science and Technology (UNCST-#SS 4283) and the London School of Hygiene and Tropical Medicine Observational / Interventions Research Ethics Committee (LSHTM Ethics #21802)

Role of the funding source

The funders had no role in study design, data collection and analysis, decision to publish or preparation of the manuscript.

Results

Of 5,812 participants in both rounds we included 5,678 (97.7%) participants with kidney function and mortality data available for the analysis (**Figure 1**). The median age of the participants was 36 years (interquartile range (IQR) 24-50 years) with most being female (60.7%). Among those with data available, 70.2 % were normal weight, 9.8% were HIV-positive, 90.5% were non-smokers, 58.5% did not consume alcohol, 10.3% were hypertensive and 1.5% of the participants were diabetic (**Table 1**). Comparison of baseline characteristics between participants eligible for inclusion from the 2011-12 and the 2014-2015 rounds is shown in **Supplementary Table 1**. We also compare baseline characteristics of participants by five-level stages of kidney function including a separate category for people with eGFR >120ml/min/1.73m^2 in **Supplementary Table 2**.

During a median follow-up of 5.0 years (IQR 3.7-6.0) there were 140 deaths. The incidence rate for participants with eGFR >90ml/min/1.73m^2, eGFR 60-90 ml/min/1.73m^2, eGFR 45-60 ml/min/1.73m^2, and eGFR <45 ml/min/1.73m^2 was 3, 9, 19, and 63 deaths per 1000 PYAR, respectively. During follow-up, 26.4% (1,500/5,678) of participants migrated and 3.0% (169/5,678) were lost to follow-up.

For peer review only - http://bmjopen.bmj.com/site/about/guidelines.xhtml

Table 1. Baseline characteristics of participants categorised by level of kidney function in the general population cohort (N=5,678)

Variable	Estimated glomerular filtration rate (mls/min/1·73m²) ≥90 N (%) 4563 (80.4)	60-89 N (%) 1022 (18.0)	45-59 N (%) 73 (1.3)	<45 N (%) 20 (0.4)	Total N (%) 5678 (100)
**Sex**					
Male	1853 (40·6)	343 (33·6)	26 (35·6)	7 (35·0)	2229 (39·3)
Female	2710 (59·4)	679 (66·4)	47 (64·4)	13 (65·0)	3449 (60·7)
**Age (years), median (IQR)**	32 (22-44)	56 (45-70)	66 (58-76)	68 (42-76)	36 (24-50)
**Age groups, N (%)**					
<35	2531 (55·5)	76 (7·4)	3 (4·1)	3 (15·0)	2613 (46·0)
35-44	946 (20·7)	176 (17·2)	4 (5·5)	2 (10·0)	1128 (19·9)
45-54	619 (13·6)	210 (20·6)	10 (13·7)	3 (15·0)	842 (14·8)
55-64	311 (6·8)	214 (20·9)	14 (19·2)	1 (5·0)	540 (9·5)
65-75	133 (2·9)	186 (18·2)	19 (26·0)	5 (25·0)	343 (6·0)
>75	23 (0·5)	160 (15·7)	23 (31·5)	6 (30·0)	212 (3·7)
**HIV status, N (%)**					
Negative	4134 (90·6)	901 (88·2)	70 (95·9)	13 (65·0)	5118 (90·1)
Positive	424 (9·3)	117 (11·5)	3 (4·1)	7 (35·0)	551 (9·7)
Missing	5 (0·1)	4 (0·4)	0 (0·0)	0 (0·0)	9 (0·2)
**BMI classification,# (kg/m²) N (%)**					
Underweight (<18·5)	504 (11·1)	143 (14·0)	14 (19·2)	4 (10·0)	665 (11·7)
Normal weight (18·5–24·99)	3201 (70·2)	623 (61·0)	41 (56·2)	14 (70·0)	3879 (68·3)
Overweight (25·0–29·99)	552 (12·1)	166 (16·2)	14 (19·2)	2 (10·0)	734 (12·9)
Obese (>30·0)	167 (3·7)	77 (7·5)	3 (4·1)	0 (0·0)	247 (4·4)
Missing	139 (3·1)	13 (1·3)	1 (1·4)	0 (0·0)	153 (2·7)
**Diabetes mellitus,## N (%)**					
No	2998 (65·7)	815 (79·8)	62 (84·9)	16 (100·0)	3891 (68·5)
Yes	62 (1·4)	20 (2·0)	3 (4·1)	0 (0·0)	85 (1·5)
Missing	1503 (32·9)	187 (18·3)	8 (11·0)	4 (20·0)	1702 (30·0)
**Hypertension,### N (%)**					
Normal	2756 (60·4)	614 (60·1)	35 (48·0)	9 (45·0)	3414 (60·1)
Hypertensive	319 (7·0)	228 (22·3)	30 (41·1)	7 (35·0)	584 (10·3)
Missing	1488 (32·6)	180 (17·6)	8 (11·0)	4 (20·0)	1680 (29·6)
**Smoking Status, N (%)**					
Not current smokers	2801 (61·4)	748 (73·2)	55 (75·3)	13 (65·0)	3617 (63·7)
Non-daily smokers	64 (1·4)	30 (2·9)	1 (1·4)	1 (5·0)	96 (1·7)
Daily smokers	209 (4·6)	64 (6·3)	9 (12·3)	2 (10·0)	284 (5·0)
Missing	1489 (32·6)	180 (17·6)	8 (11·0)	4 (20·0)	1681 (29·6)
**Alcohol Consumption, N (%)**					
Infrequent/no alcohol	1911 (41·9)	393 (38·5)	29 (39·7)	4 (20·0)	2337 (41·2)
Regular alcohol	1164 (25·5)	449 (43·9)	36 (49·3)	12 (60·0)	1661 (29·3)
Missing	1488 (32·6)	180 (17·6)	8 (11·0)	4 (20·0)	1680 (29·6)
**Marital status, N (%)**					
Single/widowed	853 (18·7)	444 (43·4)	37 (50·7)	13 (65·0)	1347 (23·7)
Married	2496 (54·7)	536 (52·5)	33 (45·2)	7 (35·0)	3072 (54·1)
Missing	1214 (26·6)	42 (4·1)	3 (4·1)	0 (0·0)	1259 (22·2)

#Body Mass Index (BMI) Classification according to WHO (weight/height²: kg/m²)
##Diabetes mellitus defined as HBA1C >6.5%, previously diagnosed with diabetes mellitus or being on treatment for diabetes
###Hypertension was defined as having a systolic BP ≥90 mmHg, diastolic BP ≥140 mmHg or being on treatment for hypertension

For peer review only

For peer review only - http://bmjopen.bmj.com/site/about/guidelines.xhtml

Age-sex and fully adjusted associations with mortality are shown in **Table 2** and **Figure 2**. In age- and sex-adjusted analyses, eGFR <45ml/min/1.73m^2 at baseline was associated with a 5.97 (95% CI 2.55-13.98) increased risk of mortality compared to those with baseline eGFR >90ml/min/1.73m^2. After inclusion of additional confounders into the model eGFR <45ml/min/1.73m^2 at baseline remained strongly associated with mortality (HR 6.12, 95% CI 2.27-16.45). The test for trend showed strong evidence (P<0.001) that the mortality rate increased progressively as category of baseline kidney function decreased.

There was no evidence of an interaction between HIV and kidney function with risk of mortality in the fully adjusted model (P=0.672).

In an additional analysis, when very high eGFR was included as a separate category there was weak evidence of a 'reverse J-shaped curve' (**Supplementary Figure 1 and Supplementary Table 3**). In age-sex adjusted analysis, baseline eGFR ≥120ml/min/1.73m^2 was associated with increased risk of mortality (HR 2.68, 95% CI 1.47-4.87) compared to the reference category of 90-119ml/min/1.73m^2. However, in fully adjusted analyses confidence intervals crossed the null (HR 1.65, 95% CI 0.61-4.44).

For peer review only - http://bmjopen.bmj.com/site/about/guidelines.xhtml

Table 2. Results of age-sex and fully adjusted regression models for the association between kidney function and mortality in the general population cohort

	Age and sex adjusted HR (95% CI) N=5678	P-value	Fully adjusted# HR (95% CI) N=3102	P-value
**eGFR (ml/min/1.73m²)**				
≥90	**Reference**		**Reference**	
60-89	1·11 (0·73-1·71)		1·21 (0·73-2·03)	
45-59	1·72 (0·81-3·69)		1·91 (0·80-4·54)	
<45	5·97 (2·55-13·98)	<0·001	6·12 (2·27-16·45)	0·003
**Age (years)**	1·06 (1·05-1·07)	<0·001	1·05 (1·03-1·07)	<0·001
**Sex**				
Male	**Reference**		**Reference**	
Female	0·50 (0·36-0·70)	<0·001	0·70 (0·42-1·07)	0·188
**HIV status**				
Positive			1·67 (0·87-3·18)	0·120
Negative			**Reference**	
**Hypertension**				
Normotensive			**Reference**	
Hypertensive			1·08 (0·58-2·01)	0·433
**Diabetes mellitus**				
No			**Reference**	
Yes			1·11 (0·35-3·57)	0·861
**BMI classification**				
Under weight			1·71 (1·10-2·67)	
Normal weight			**Reference**	
Overweight			0·20 (0·05-0·84)	
Obese			0·35 (0·48-2·55)	0·006
**Marital status**				
Currently married			0·77 (0·48-1·23)	0·273
Single/widowed			**Reference**	
**Alcohol consumption**				
Regular alcohol			1·50 (0·92-2·46)	0·104
No/infrequent alcohol			**Reference**	
**Smoking status**				
Not current smokers			**Reference**	
Non-daily smokers			1·22 (0·50-2·97)	
Daily smokers			1·63 (0·93-2·87)	0·236

#Fully Adjusted Hazard Ratios adjusted for age, sex, HIV status, hypertension, diabetes mellitus, BMI, marital status, alcohol use and smoking.

For peer review only - http://bmjopen.bmj.com/site/about/guidelines.xhtml

Discussion

In a rural Ugandan population cohort, we found a graded association between low baseline kidney function and subsequent mortality. Participants with severe kidney impairment (eGFR <45ml/min/1.73m^2) had a more than six-fold risk of dying compared to those with eGFR >90ml/min/1.73m^2. This was despite a low prevalence of obesity, diabetes and regular smoking, all key risk factors for kidney disease in high-income countries.

Our study has a number of strengths. It is the first data of its kind from sub-Saharan Africa, generated in a large, well-established population cohort with robust standardised procedures for detailed measurements of covariates such as blood pressure, and creatinine was measured according to recommended standards.[13] There is high participation and retention in the study within the local community and regular reporting of mortality and migration by local study workers leading to limited loss to follow-up.[13]

However, there were also limitations. This cohort covers a region that is mostly rural and may not be generalisable to urban regions. However, our study population is in keeping with the majority of people living in Uganda and in many regions of sub-Saharan Africa. There was missing data for covariates such as smoking, diabetes and blood pressure for about one-third of participants so the complete case analysis led to reduction in power for the fully adjusted model. In addition, participants in the 2011-12 round, who were more likely to have had complete data for potential confounders, also had longer follow-up time over which they could have died. However, marginal differences between the effect estimates for the association of eGFR categories with mortality between the age-sex adjusted and fully adjusted models suggests that neither confounding nor selection bias due to variable follow-up have substantially impacted on our findings of the association between baseline kidney function and mortality. We used baseline measures of covariates and were not able to update health related confounders over time. There is likely to be inaccuracy in measurement of kidney function which we categorised using estimated GFR calculated from serum creatinine, a breakdown product of muscle and related to muscle mass and dietary meat

For peer review only - http://bmjopen.bmj.com/site/about/guidelines.xhtml

intake.[16] Therefore, the prevalence of low BMI in this population where food scarcity is common[21] means that we may have underestimated the prevalence of impaired GFR in this population. In addition, validation of GFR estimating equations is currently limited in sub-Saharan Africa.[8] We measured kidney function only once while two measures 3 months apart are required to confirm a diagnosis of chronic kidney disease which may have led to misclassification of level of kidney function for some participants.[18] In addition, we do not have measures of proteinuria, an important early marker of kidney damage so cannot comment on the association with mortality in this setting. Migration and subsequent loss to follow-up among younger, healthier participants may have led to selection bias with overrepresentation of older participants with health problems including impaired kidney function remaining in the cohort. However, people with chronic health problems often return home so we anticipate capturing the majority of deaths among the baseline cohort, even among those who previously migrated. Finally, we do not know the causes of death, and small numbers of people in lower kidney function subgroups led to limited power and imprecise estimates with wide confidence intervals.

Recent community-based studies of associations of kidney disease suggest important differences in prevalence and associations of impaired kidney function between countries in sub-Saharan Africa. Those including participants from urban areas in South Africa and Nairobi found similar risk factors for those well-established in high-income countries: age, hypertension, diabetes, HIV and female sex.[22] However, cohorts in Malawi and Uganda where the populations are younger, often living in rural areas and with low levels of smoking, obesity and diabetes showed low prevalence of impaired kidney function and found an association with diabetes, possibly due to hyperfiltration.[14 23] These findings suggest that the aetiology of kidney disease in rural sub-Saharan Africa is unclear, and highlight the possibility that the mechanism by which kidney function is associated with increased mortality may differ from that in high-income countries.

For peer review only - http://bmjopen.bmj.com/site/about/guidelines.xhtml

For peer review only

Prospective studies that have looked at the association between mortality and kidney disease in sub-Saharan Africa are limited to cohorts which are hospital based,[24-27] include patients with only ESKD[28] or HIV,[29] study albuminuria alone,[30] and many are also limited to short-term follow-up. Our study is consistent with results from high-income countries which have shown a consistent, strong and graded association between impaired kidney function and risk of subsequent mortality.[31] These studies also demonstrate an increase in mortality with people at the highest level of eGFR, in a reverse 'J-shaped curve'. Our findings are again consistent, demonstrating weak evidence of an increase in mortality for people with the highest category of baseline eGFR. Further confirmation will be needed in larger cohorts with longer follow-up.

While HIV can cause kidney disease[32] we did not find an interaction between HIV, impaired kidney function and mortality, suggesting that low kidney function has the same pattern of association with mortality among people who are HIV negative. Unexpectedly, we did not find an association between hypertension and mortality in our study despite evidence that it is a key driver of disease and outcomes in sub-Saharan Africa and globally.[33] It is possible that repeated screening within this cohort population has led to higher rates of treatment and control than in other populations, and prevalence of hypertension in this region (10.3%) was lower than the estimated national prevalence of 26.4%.[34] However, it is also possible that the causal relationship between hypertension and kidney damage in sub-Saharan Africa has been oversimplified due to lack of kidney function measurement and longitudinal follow-up. Recent discovery of strong genetic risks for kidney disease in African-Americans has suggested that hypertension and kidney dysfunction may develop in part from shared risk factors.[35] Similarly, diabetes is an independent predictor of mortality among patients with kidney disease and is the leading cause of CKD globally.[36] However, in this population with a low prevalence of diabetes it was not associated with mortality.

In conclusion, in this prospective cohort study based in a rural Ugandan community we found that baseline impaired kidney function was associated with mortality, which remained after adjusting for

For peer review only - http://bmjopen.bmj.com/site/about/guidelines.xhtml

known shared risk factors such as diabetes, hypertension and tobacco and despite the co-existent burden of high infectious disease mortality. This suggests that kidney function plays a key role in overall health status in sub-Saharan Africa and, since options for treatment of kidney failure are very restricted, should be included within public health targets. Improved understanding of the determinants of kidney disease and its progression are needed in order to inform interventions for prevention and treatment.

For peer review only

For peer review only - http://bmjopen.bmj.com/site/about/guidelines.xhtml

Acknowledgement

We thank Rose Nabwato (Study Nurse), Josephine Kagina Nabukenya (Study Nurse), Vincent Arumadri (Laboratory Technician), Francis Namugera (Data Clerk), Eva Nambi Ssejjemba (Study Administrator) and Grace Godfrey Sseremba (Community Mobilizer) for the role they played in the study.

Author Contributions Statement

RK conceived the idea. RK, KT, JS and RN and CHH advised on methodology. JS, RN, LS and LAT obtained the funding. RK, KT, BS, RM, MKM, JS and RN collected and curated the data and administered the project. RK and IS analysed the data. RK and LAT wrote the original draft and all authors contributed to and approved the final manuscript.

Additional Information

Collaborators Statement

IS and KT have verified the underlying data. All authors confirm that they had full access to all the data in the study and accept responsibility to submit for publication.

Declaration of interests

The authors declare that there are no conflicts of interest

Data sharing

Owing to data protection concerns, there are restrictions on access to the underlying data. The GPC database contains 25 years of longitudinal data sets on demographics and disease surveillance. All data (census, survey and laboratory) generated through the cohort are stored and curated at the MRC/UVRI and the LSHTM Research Unit. Data access for specific research purposes is possible and has been granted previously. For any data access inquiries, you may contact the director, MRC/UVRI and the LSHTM Research Unit or by email to mrc@mrcuganda.org or the corresponding author.

Funding

This work was funded by GlaxoSmithKline Africa Non-Communicable Disease Open Lab grant number: 8111 as part of a broader multicenter collaborative study between South Africa, Uganda, Malawi and the London School of Hygiene and Tropical Medicine which is collectively identified as the African Research in Kidney Disease (ARK) Network. This work was also funded by Wellcome Trust intermediate clinical fellowship grant number 101143/Z/13/Z awarded to LAT. The General population cohort is funded by the UK Medical Research Council (MRC) and the UK Department for International Development (DFID) under the MRC/DFID Concordat agreement and is also part of the

For peer review only - http://bmjopen.bmj.com/site/about/guidelines.xhtml

EDCTP2 programme supported by the European Union grant number not applicable. This work was also funded by THRiVE-2, a DELTAS Africa grant number DEL-15-011 from Wellcome Trust grant number 107742/Z/15/Z and the UK government which supported JS.

Figure 1: Title: Study Flow Chart

For peer review only

For peer review only - http://bmjopen.bmj.com/site/about/guidelines.xhtml

Figure 2: Hazard ratios and 95% CI for the fully adjusted associations of baseline eGFR and mortality in a rural Ugandan population cohort

For peer review only

For peer review only - http://bmjopen.bmj.com/site/about/guidelines.xhtml

References

1. Stanifer JW, Jing B, Tolan S, et al. The epidemiology of chronic kidney disease in sub-Saharan Africa: a systematic review and meta-analysis. *Lancet Glob Health* 2014;2(3):e174-81. doi: 10.1016/S2214-109X(14)70002-6
2. Collaboration GBDCKD. Global, regional, and national burden of chronic kidney disease, 1990-2017: a systematic analysis for the Global Burden of Disease Study 2017. *Lancet* 2020;395(10225):709-33. doi: 10.1016/S0140-6736(20)30045-3 [published Online First: 2020/02/18]
3. Wang HE, Gamboa C, Warnock DG, et al. Chronic kidney disease and risk of death from infection. *Am J Nephrol* 2011;34(4):330-6. doi: 10.1159/000330673 [published Online First: 2011/08/24]
4. Sarnak MJ, Levey AS, Schoolwerth AC, et al. Kidney disease as a risk factor for development of cardiovascular disease: a statement from the American Heart Association Councils on Kidney in Cardiovascular Disease, High Blood Pressure Research, Clinical Cardiology, and Epidemiology and Prevention. *Hypertension* 2003;42(5):1050-65. doi: 10.1161/01.HYP.0000102971.85504.7c [published Online First: 2003/11/08]
5. Thurlow JS, Joshi M, Yan G, et al. Global Epidemiology of End-Stage Kidney Disease and Disparities in Kidney Replacement Therapy. *Am J Nephrol* 2021;52(2):98-107. doi: 10.1159/000514550 [published Online First: 2021/03/23]
6. Liyanage T, Ninomiya T, Jha V, et al. Worldwide access to treatment for end-stage kidney disease: a systematic review. *Lancet* 2015;385(9981):1975-82. doi: 10.1016/S0140-6736(14)61601-9 [published Online First: 2015/03/18]
7. Kalyesubula R, Mutyaba I, Rabin T, et al. Trends of admissions and case fatality rates among medical in-patients at a tertiary hospital in Uganda; A four-year retrospective study. *PloS one* 2019;14(5):e0216060. doi: 10.1371/journal.pone.0216060 [published Online First: 2019/05/16]
8. Fabian J, George JA, Etheredge HR, et al. Methods and reporting of kidney function: a systematic review of studies from sub-Saharan Africa. *Clin Kidney J* 2019;12(6):778-87. doi: 10.1093/ckj/sfz089 [published Online First: 2019/12/07]
9. Delanaye P, Ebert N, Pottel H. Newer GFR Estimating Equations Require Validation in Different Populations. *American journal of kidney diseases : the official journal of the National Kidney Foundation* 2017;70(4):586. doi: 10.1053/j.ajkd.2017.06.016
10. Delanaye P, Mariat C, Maillard N, et al. Are the creatinine-based equations accurate to estimate glomerular filtration rate in African American populations? *Clinical journal of the American Society of Nephrology : CJASN* 2011;6(4):906-12. doi: 10.2215/CJN.10931210
11. Levey AS, Stevens LA, Schmid CH, et al. A new equation to estimate glomerular filtration rate. *Annals of internal medicine* 2009;150(9):604-12.
12. Nations U. World Bank staff estimates based on the United Nations Population Division's World Urbanization Prospects: 2018 Revision. United Nations. New York: United Nations, 2019:https://population.un.org/wup/Publications/Files/WUP2018-Report.pdf.
13. Asiki G, Murphy G, Nakiyingi-Miiro J, et al. The general population cohort in rural south-western Uganda: a platform for communicable and non-communicable disease studies. *International journal of epidemiology* 2013;42(1):129-41. doi: 10.1093/ije/dys234
14. Kalyesubula R, Hau JP, Asiki G, et al. Impaired renal function in a rural Ugandan population cohort. *Wellcome open research* 2018;3:149. doi: 10.12688/wellcomeopenres.14863.3
15. Galiwango RM, Musoke R, Lubyayi L, et al. Evaluation of current rapid HIV test algorithms in Rakai, Uganda. *J Virol Methods* 2013;192(1-2):25-7. doi: 10.1016/j.jviromet.2013.04.003 [published Online First: 2013/04/16]
16. Myers GL, Miller WG, Coresh J, et al. Recommendations for improving serum creatinine measurement: a report from the Laboratory Working Group of the National Kidney Disease

For peer review only - http://bmjopen.bmj.com/site/about/guidelines.xhtml

Education Program. *Clin Chem* 2006;52(1):5-18. doi: 10.1373/clinchem.2005.0525144 [published Online First: 2005/12/08]

17. Bukabau JB, Sumaili EK, Cavalier E, et al. Performance of glomerular filtration rate estimation equations in Congolese healthy adults: The inopportunity of the ethnic correction. *PloS one* 2018;13(3):e0193384. doi: 10.1371/journal.pone.0193384
18. Inker LA, Astor BC, Fox CH, et al. KDOQI US commentary on the 2012 KDIGO clinical practice guideline for the evaluation and management of CKD. *Am J Kidney Dis* 2014;63(5):713-35. doi: 10.1053/j.ajkd.2014.01.416 [published Online First: 2014/03/22]
19. Murray CJ, Lopez AD, Feehan DM, et al. Validation of the symptom pattern method for analyzing verbal autopsy data. *PLoS Med* 2007;4(11):e327. doi: 10.1371/journal.pmed.0040327 [published Online First: 2007/11/23]
20. Babua C, Kalyesubula R, Okello E, et al. Cardiovascular risk factors among patients with chronic kidney disease attending a tertiary hospital in Uganda. *Cardiovasc J Afr* 2015;26(4):177-80. doi: 10.5830/CVJA-2015-045 [published Online First: 2015/09/26]
21. Frelat R, Lopez-Ridaura S, Giller KE, et al. Drivers of household food availability in sub-Saharan Africa based on big data from small farms. *Proc Natl Acad Sci U S A* 2016;113(2):458-63. doi: 10.1073/pnas.1518384112 [published Online First: 2015/12/30]
22. George JA, Brandenburg JT, Fabian J, et al. Kidney damage and associated risk factors in rural and urban sub-Saharan Africa (AWI-Gen): a cross-sectional population study. *Lancet Glob Health* 2019;7(12):e1632-e43. doi: 10.1016/S2214-109X(19)30443-7 [published Online First: 2019/11/12]
23. Nakanga WP, Prynn JE, Banda L, et al. Prevalence of impaired renal function among rural and urban populations: findings of a cross-sectional study in Malawi. *Wellcome Open Res* 2019;4:92. doi: 10.12688/wellcomeopenres.15255.1 [published Online First: 2019/10/28]
24. Conroy AL, Opoka RO, Bangirana P, et al. Acute kidney injury is associated with impaired cognition and chronic kidney disease in a prospective cohort of children with severe malaria. *BMC Med* 2019;17(1):98. doi: 10.1186/s12916-019-1332-7 [published Online First: 2019/05/22]
25. Dlamini TAL, Heering PJ, Chivese T, et al. A prospective study of the demographics, management and outcome of patients with acute kidney injury in Cape Town, South Africa. *PloS one* 2017;12(6):e0177460. doi: 10.1371/journal.pone.0177460 [published Online First: 2017/06/02]
26. Bagasha P, Nakwagala F, Kwizera A, et al. Acute kidney injury among adult patients with sepsis in a low-income country: clinical patterns and short-term outcomes. *BMC nephrology* 2015;16:4. doi: 10.1186/1471-2369-16-4 [published Online First: 2015/01/17]
27. Olowu WA, Niang A, Osafo C, et al. Outcomes of acute kidney injury in children and adults in sub-Saharan Africa: a systematic review. *Lancet Glob Health* 2016;4(4):e242-50. doi: 10.1016/S2214-109X(15)00322-8 [published Online First: 2016/03/26]
28. Ashuntantang G, Osafo C, Olowu WA, et al. Outcomes in adults and children with end-stage kidney disease requiring dialysis in sub-Saharan Africa: a systematic review. *Lancet Glob Health* 2017;5(4):e408-e17. doi: 10.1016/S2214-109X(17)30057-8 [published Online First: 2017/02/24]
29. Mulenga LB, Kruse G, Lakhi S, et al. Baseline renal insufficiency and risk of death among HIV-infected adults on antiretroviral therapy in Lusaka, Zambia. *Aids* 2008;22(14):1821-7. doi: 10.1097/QAD.0b013e328307a051
30. Schutte R, Schmieder RE, Huisman HW, et al. Urinary albumin excretion from spot urine samples predict all-cause and stroke mortality in Africans. *Am J Hypertens* 2014;27(6):811-8. doi: 10.1093/ajh/hpt288 [published Online First: 2014/02/12]
31. Chronic Kidney Disease Prognosis C, Matsushita K, van der Velde M, et al. Association of estimated glomerular filtration rate and albuminuria with all-cause and cardiovascular mortality in general population cohorts: a collaborative meta-analysis. *Lancet*

For peer review only - http://bmjopen.bmj.com/site/about/guidelines.xhtml

2010;375(9731):2073-81. doi: 10.1016/S0140-6736(10)60674-5 [published Online First: 2010/05/21]
32. Swanepoel CR, Atta MG, D'Agati VD, et al. Kidney disease in the setting of HIV infection: conclusions from a Kidney Disease: Improving Global Outcomes (KDIGO) Controversies Conference. *Kidney international* 2018;93(3):545-59. doi: 10.1016/j.kint.2017.11.007 [published Online First: 2018/02/06]
33. Plange-Rhule J, Phillips R, Acheampong JW, et al. Hypertension and renal failure in Kumasi, Ghana. *J Hum Hypertens* 1999;13(1):37-40. doi: 10.1038/sj.jhh.1000726 [published Online First: 1999/02/03]
34. Guwatudde D, Mutungi G, Wesonga R, et al. The Epidemiology of Hypertension in Uganda: Findings from the National Non-Communicable Diseases Risk Factor Survey. *PloS one* 2015;10(9):e0138991. doi: 10.1371/journal.pone.0138991 [published Online First: 2015/09/26]
35. Nadkarni GN, Coca SG. APOL1 and blood pressure changes in young adults. *Kidney Int* 2017;92(4):793-95. doi: 10.1016/j.kint.2017.05.030 [published Online First: 2017/09/25]
36. Alicic RZ, Rooney MT, Tuttle KR. Diabetic Kidney Disease: Challenges, Progress, and Possibilities. *Clin J Am Soc Nephrol* 2017;12(12):2032-45. doi: 10.2215/CJN.11491116 [published Online First: 2017/05/20]

For peer review only

For peer review only - http://bmjopen.bmj.com/site/about/guidelines.xhtml

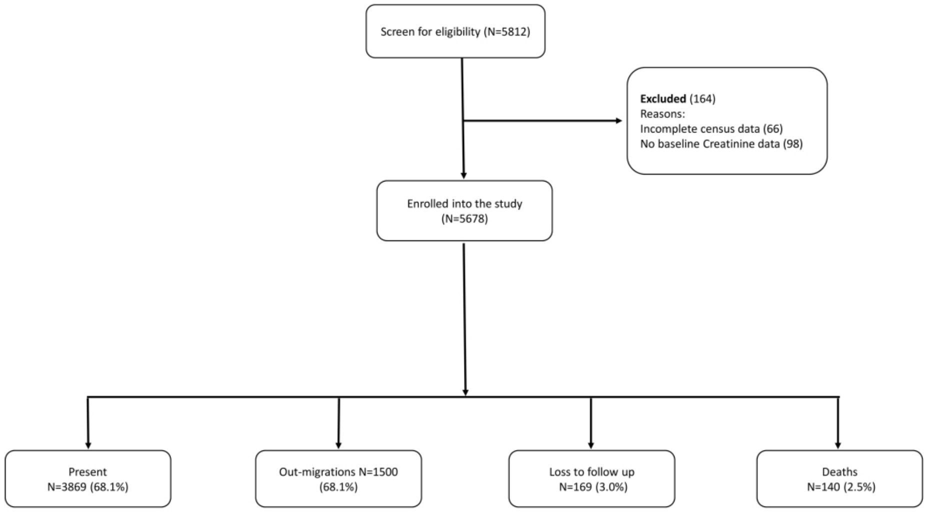

Study Flow Diagram

338x190mm (300 x 300 DPI)

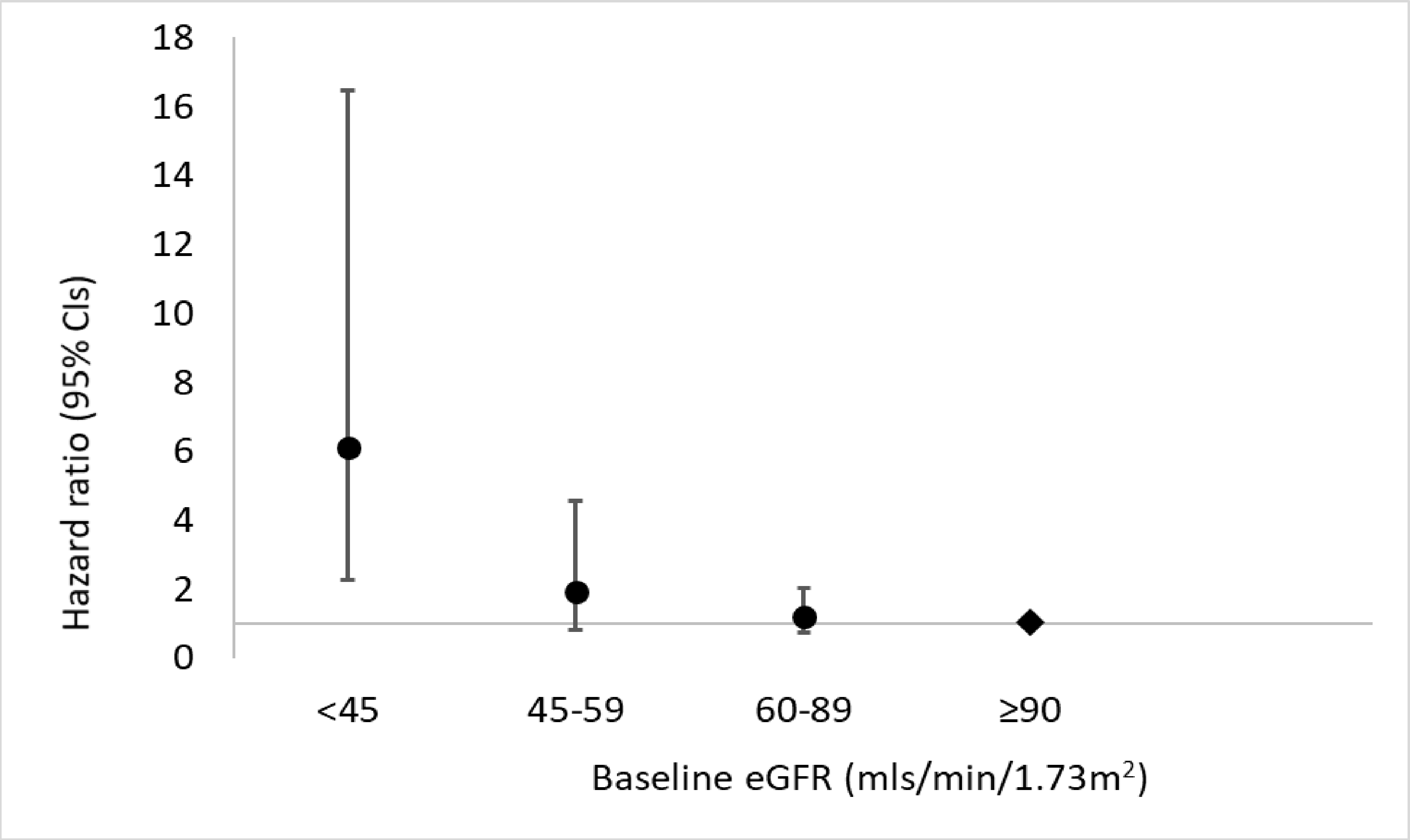
18
16
14
12
10
8
6
4
2
0
Hazard ratio (95% CIs)
<45
45-59
60-89
≥90
Baseline eGFR (mls/min/1.73m2)

Supplementary Appendix for

"Association of impaired kidney function with mortality in rural Uganda: results of a general population cohort study"

For peer review only

For peer review only - http://bmjopen.bmj.com/site/about/guidelines.xhtml

Supplementary Table 1. Comparison between participants included and excluded from the main analysis

Variable	Participants with complete data included in fully adjusted model N=3102	Participants excluded from the final model N=2567	P-value
**eGFR (ml/min/1.73m²)**			
≥90	2231 (71·9)	2332 (90.5)	
60-89	792 (25·6)	230 (8.9)	
45-59	63 (2·0)	10 (0.4)	
<45	16 (0·5)	4 (0·2)	<0.001
**Gender**			
Male	1150 (37·1)	1079 (41.9)	
Female	1952 (62·9)	1497 (58.1)	<0.001
**Age groups, N (%)**			
<35	733 (23·6)	1880 (73.0)	
35-44	772 (24·9)	356 (13.8)	
45-54	682 (22·0)	160 (6.2)	
55-64	451 (14·5)	89 (3.5)	
65-75	291 (9·4)	52 (2.0)	
>75	173 (5·6)	39 (1.5)	<0.001
**Age (years), median (IQR)**	45 (35-57)	24 (19-36)	<0.001
**HIV status, **N (%)**			
Negative	2756 (88·8)	2362 (91.7)	
Positive	346 (11·2)	205 (8.0)	
Missing	··	9 (0·3)	<0.001
**BMI classification, (kg/m²) # N (%)**			
Underweight (<18·5)	415 (13·4)	250 (9.7)	
Normal weight (18·5–24·99)	2029 (65·4)	1850 (71.8)	
Overweight (25·0–29·99)	484 (15·6)	250 (9.7)	
Obese (>30·0)	174 (5·6)	73 (2.8)	
Missing	··	153 (5.9)	<0.001
**Diabetes mellitus, ## N (%)**			
No	3025 (97·5)	866 (33.6)	
Yes	77 (2·5)	8 (0.3)	
Missing	··	1702 (66.1)	<0.001
**Hypertension, ### N (%)**			
Normal	2583 (83·3)	831 (32.3)	
Hypertensive	519 (16·7)	65 (2.5))	
Missing	··	1680 (65.2)	<0.001
**Smoking Status, N (%)**			
Not current smokers	2757 (88·9)	860 (33.4)	
Non-daily smokers	79 (2·6)	17 (0.7)	
Daily smokers	266 (8·5)	18 (0.7)	
Missing	··	1681 (65.3)	<0.001
**Alcohol Consumption, N (%)**			
Regular alcohol	1601 (51·6)	736 (28.6)	
Infrequent/no alcohol	1501 (48·4)	160 (6.2)	
Missing	··	1680 (65.2)	<0.001
**Currently married, N(%)**			
Married	978 (31·5)	369 (14.3)	
Single/widowed	2124 (68·5)	948 (36.8)	
Missing	··	1259 (48.9)	<0.001

[#]Body Mass Index (BMI) Classification according to WHO (weight/height²: kg/m²)
[##]Diabetes mellitus defined as HBA1C >6.5%, previously diagnosed with diabetes mellitus or being on treatment for diabetes
[###]Hypertension was defined as having a systolic BP ≥90 mmHg, diastolic BP ≥140 mmHg or being on treatment for hypertension

For peer review only

Supplementary Table 2. Baseline characteristics of participants by five-level categories of kidney function in the general population cohort (n=5,678)

	Estimated glomerular filtration rate (mls/min/1.73m²)					
	≥120	90-119	60-89	45-59	<45	Total
Variable	N%	N%	N%	N%	N%	
	1966 (34.6)	2597 (45.7)	1022 (18.0)	73 (1.3)	20 (0.4)	
**Sex**						
Male	709 (36·1)	1144 (44·1)	343 (33·6)	26 (35·6)	7 (35·0)	2229 (39·3)
Female	1257 (63·9)	1453 (56·0)	679 (66·4)	47 (64·4)	13 (65·0)	3449 (60·7)
**Age (years), median (IQR)**	22 (19-28)	41 (33-51)	56 (45-70)	66 (58-76)	68 (42-76)	36 (24-50)
**Age groups, N (%)**						
<35	1784 (90·7)	747 (28·8)	76 (7·4)	3 (4·1)	3 (15·0)	2613 (46·0)
35-44	158 (8·0)	788 (30·3)	176 (17·2)	4 (5·5)	2 (10·0)	1128 (19·9)
45-54	17 (0·9)	602 (23·2)	210 (20·6)	10 (13·7)	3 (15·0)	842 (14·8)
55-64	5 (0·3)	306 (11·8)	214 (20·9)	14 (19·2)	1 (5·0)	540 (9·5)
65-75	2 (0·1)	131 (5·0)	186 (18·2)	19 (26·0)	5 (25·0)	343 (6·0)
>75	0 (0·0)	23 (0·9)	160 (15·7)	23 (31·5)	6 (30·0)	212 (3·7)
**HIV status, N (%)**						
Negative	1836 (93·4)	2298 (88·5)	901 (88·2)	70 (96·0)	13 (65·0)	5118 (90·1)
Positive	127 (6·5)	297 (11·4)	117 (11·5)	3 (4·1)	7 (35·0)	551 (9·7)
Missing	3 (0·2)	2 (0·1)	4 (0·4)	0 (0·0)	0 (0·0)	9 (0·2)
**BMI classification, # (kg/m²) N (%)**						
Underweight (<18·5)	185 (9·4)	319 (12·3)	143 (14·0)	14 (19·2)	4 (20·0)	665 (11·7)
Normal weight (18·5–<25)	1429 (72·7)	1772 (68·2)	623 (61·0)	41 (56·2)	14 (70·0)	3879 (68·3)
Overweight (25·0–<30)	195 (9·9)	357 (13·8)	166 (16·2)	14 (19·2)	2 (10·0)	734 (12·9)
Obese (≥30·0)	46 (2·3)	121 (4·7)	77 (7·6)	3 (4·1)	0 (0·0)	247 (4·4)
Missing	111 (5·7)	28 (1·1)	13 (1·3)	1 (1·4)	0 (0)	153 (2·7)
**Diabetes mellitus, ## N (%)**						
No	1096 (55·8)	1902 (73·2)	815 (79·8)	62 (84·9)	16 (80·0)	3926 (68·5)
Yes	13 (0·7)	49 (1·9)	20 (2·0)	3 (4·1)	0 (0·0)	85 (1·5)
Missing	857 (43·6)	646 (24·9)	187(18·3)	8 (11·0)	4 (20·0)	1702 (30·0)
**Hypertension, ### N (%)**						
Normal	1064 (54·1)	1692 (65·2)	614 (60·1)	35 (47·9)	9 (45·0)	3414 (60·1)
Hypertensive	48 (2·4)	271 (10·4)	228 (22·3)	30 (41·1)	7 (35·0)	584 (10·3)
Missing	854 (43·4)	634 (24·4)	180 (17·6)	8 (11·0)	4 (20·0)	1680 (29·6)
**Smoking Status, N (%)**						
Not current smokers	1061 (54·0)	1740 (67·0)	748 (73·2)	55 (75·3)	13 (65·0)	3617 (63·7)
Non-daily smokers	13 (0·7)	51 (2·0)	30 (2·9)	1 (1·4)	1 (5·0)	96 (1·7)
Daily smokers	38 (1·9)	171 (6·6)	64 (6·3)	9 (12·3)	2 (10·0)	284 (5·0)
Missing	854 (43·4)	635 (24·5)	180 (17·6)	8 (11·0)	4 (20·0)	1681 (29·6)
**Alcohol Consumption, N (%)**						
No	854 (43·4)	1057 (40·7)	393 (38·5)	29 (39·7)	4 (20·0)	2337 (41·2)
Yes	258 (13·1)	906 (34·9)	449 (43·9)	36 (49·3)	12 (60·0)	1661 (29·3)
Missing	854 (43·4)	634 (24·4)	180 (17·6)	8 (11·0)	4 (20·0)	1680 (29·6)
**Marital status, N (%)**						
Married	162 (8·2)	691 (26·6)	444 (43·4)	37 (50·7)	13 (65·0)	1347 (23·7)
Single/widowed	888 (45·2)	1608 (61·9)	536 (52·5)	33 (45·2)	7 (35·0)	3072 (54·1)
Missing	916 (46·6)	298 (11·5)	42 (4·1)	3 (4·1)	0 (0·0)	1259 (22·2)

#Body Mass Index (BMI) Classification according to WHO (weight/height²: kg/m²)
##Diabetes mellitus defined as HBA1C >6.5%, previously diagnosed with diabetes mellitus or being on treatment for diabetes
###Hypertension was defined as having a systolic BP ≥90 mmHg, diastolic BP ≥140 mmHg or being on treatment for hypertension

For peer review only - http://bmjopen.bmj.com/site/about/guidelines.xhtml

Supplementary Table 3. Results of age-sex and fully adjusted regression models for the association between five category kidney function and mortality in the general population cohort

	Age and sex adjusted		Fully adjusted#	
	**HR (95% CI) N=5678**	**P-value**	**HR (95% CI) N=3102**	**P-value**
**eGFR (mls/min/1.73m²)**				
≥120	2·68 (1·47-4·87)		1·65 (0·61-4·44)	
90-119	**reference**		**reference**	
60-89	1·14 (0·73-1·77)		1·23 (0·73-2·06)	
45-59	1·67 (0·77-3·60)		1·90 (0·79-4·52)	
<45	5·58 (2·36-13·24)	<0·001	6·06 (2·25-16·37)	0·005
**Age (years)**	1·06 (1·05-1·07)	<0·001	1·06 (1·04-1·08)	<0·001
**Sex**				
Male	**reference**		**reference**	
Female	0·50 (0·36-0·70)	<0·001	0·71 (0·42-1·19)	0·19
**HIV status**				
Positive			1·69 (0·89-3·23)	0·11
Negative				
**Hypertension**				
Normotensive			**reference**	
Hypertensive			0·82 (0·51-1·33)	0·42
**Diabetes mellitus**				
No			**reference**	
Yes			1·11 (0·34-3·56)	0·87
**BMI classification**				
Under weight			1·70 (1·09-2·66)	
Normal weight			**reference**	
Overweight			0·20 (0·05-0·84)	
Obese			0·35 (0·05-2·58)	0·006
**Marital status**				
Married			0·77 (0·48-1·22)	0·27
Single/widowed			**reference**	
**Alcohol consumption**				
Regular alcohol			1·50 (0·92-2·45)	0·11
Infrequent/no alcohol			**reference**	
**Smoking status**				
Not current smokers			**reference**	
Non-daily smokers			1·23 (0·51 - 3·00)	
Daily smokers			1·63 (0·92 - 2·86)	0·24

Fully Adjusted Hazard Ratios adjusted for age, sex, HIV status, hypertension, diabetes mellitus, BMI, marital status, alcohol use and smoking.

For peer review only - http://bmjopen.bmj.com/site/about/guidelines.xhtml

Supplementary Figure 1: Hazard ratios and 95% CI for the fully adjusted associations of baseline eGFR (with eGFR >120mls/min as a separate category) and mortality in a rural Uganda population cohort

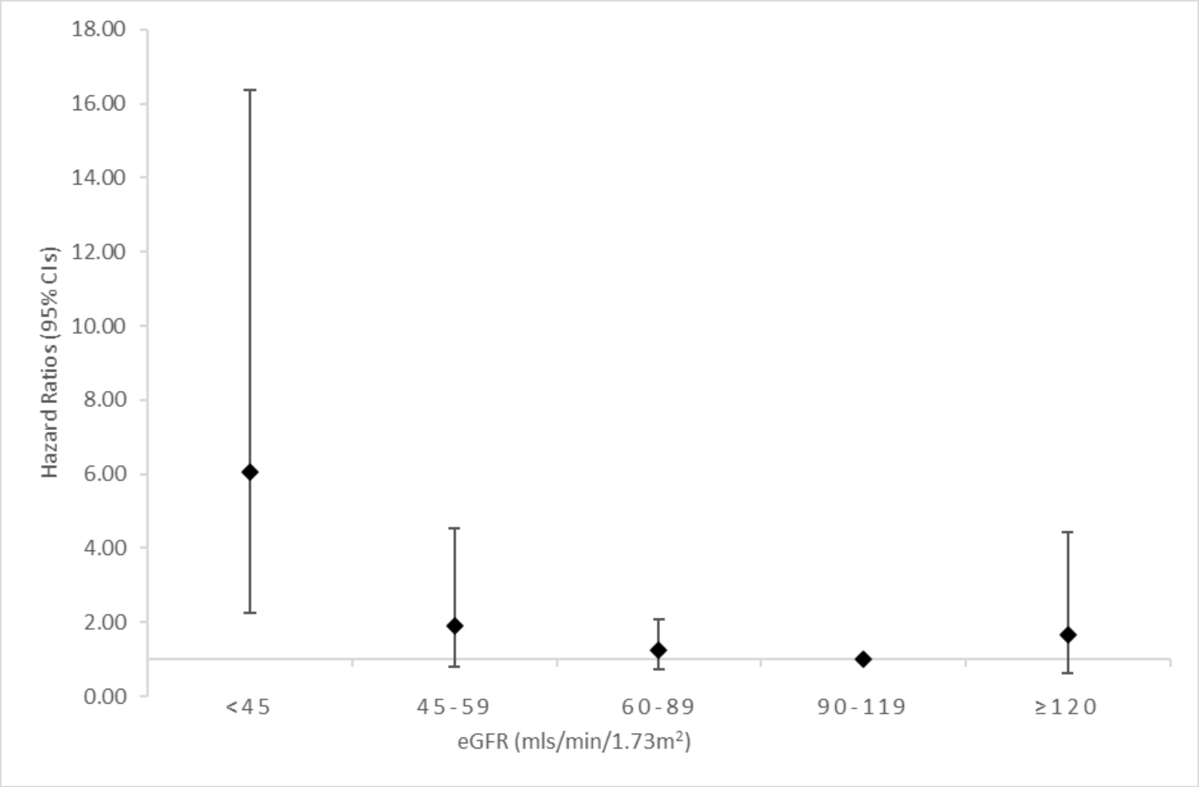

For peer review only - http://bmjopen.bmj.com/site/about/guidelines.xhtml

STROBE Statement—Checklist of items that should be included in reports of ***cohort studies***

	Item No	Recommendation	Page No
**Title and abstract**	1	(*a*) Indicate the study's design with a commonly used term in the title or the abstract	1
		(*b*) Provide in the abstract an informative and balanced summary of what was done and what was found	2
**Introduction**			
Background/rationale	2	Explain the scientific background and rationale for the investigation being reported	3
Objectives	3	State specific objectives, including any prespecified hypotheses	4
**Methods**			
Study design	4	Present key elements of study design early in the paper	4
Setting	5	Describe the setting, locations, and relevant dates, including periods of recruitment, exposure, follow-up, and data collection	4,7
Participants	6	(*a*) Give the eligibility criteria, and the sources and methods of selection of participants. Describe methods of follow-up	5
		(*b*) For matched studies, give matching criteria and number of exposed and unexposed	
Variables	7	Clearly define all outcomes, exposures, predictors, potential confounders, and effect modifiers. Give diagnostic criteria, if applicable	6
Data sources/ measurement	8*	For each variable of interest, give sources of data and details of methods of assessment (measurement). Describe comparability of assessment methods if there is more than one group	6
Bias	9	Describe any efforts to address potential sources of bias	7
Study size	10	Explain how the study size was arrived at	6
Quantitative variables	11	Explain how quantitative variables were handled in the analyses. If applicable, describe which groupings were chosen and why	6,7
Statistical methods	12	(*a*) Describe all statistical methods, including those used to control for confounding	6,7
		(*b*) Describe any methods used to examine subgroups and interactions	
		(*c*) Explain how missing data were addressed	
		(*d*) If applicable, explain how loss to follow-up was addressed	
		(*e*) Describe any sensitivity analyses	
**Results**			
Participants	13*	(a) Report numbers of individuals at each stage of study—eg numbers potentially eligible, examined for eligibility, confirmed eligible, included in the study, completing follow-up, and analysed	8
		(b) Give reasons for non-participation at each stage	8
		(c) Consider use of a flow diagram	8
Descriptive data	14*	(a) Give characteristics of study participants (eg demographic, clinical, social) and information on exposures and potential confounders	9
		(b) Indicate number of participants with missing data for each variable of interest	9
		(c) Summarise follow-up time (eg, average and total amount)	8,10
Outcome data	15*	Report numbers of outcome events or summary measures over time	8,10

For peer review only

Main results	16	(*a*) Give unadjusted estimates and, if applicable, confounder-adjusted estimates and their precision (eg, 95% confidence interval). Make clear which confounders were adjusted for and why they were included	10,11
		(*b*) Report category boundaries when continuous variables were categorized	
		(*c*) If relevant, consider translating estimates of relative risk into absolute risk for a meaningful time period	8,10
Other analyses	17	Report other analyses done—eg analyses of subgroups and interactions, and sensitivity analyses	10
**Discussion**			
Key results	18	Summarise key results with reference to study objectives	12
Limitations	19	Discuss limitations of the study, taking into account sources of potential bias or imprecision. Discuss both direction and magnitude of any potential bias	12
Interpretation	20	Give a cautious overall interpretation of results considering objectives, limitations, multiplicity of analyses, results from similar studies, and other relevant evidence	12,13,14
Generalisability	21	Discuss the generalisability (external validity) of the study results	14
**Other information**			
Funding	22	Give the source of funding and the role of the funders for the present study and, if applicable, for the original study on which the present article is based	15

*Give information separately for exposed and unexposed groups.

Note: An Explanation and Elaboration article discusses each checklist item and gives methodological background and published examples of transparent reporting. The STROBE checklist is best used in conjunction with this article (freely available on the Web sites of PLoS Medicine at http://www.plosmedicine.org/, Annals of Internal Medicine at http://www.annals.org/, and Epidemiology at http://www.epidem.com/). Information on the STROBE Initiative is available at http://www.strobe-statement.org.

For peer review only

For peer review only - http://bmjopen.bmj.com/site/about/guidelines.xhtml
